# Representation of Molecules by Sequences of Instructions

**DOI:** 10.1021/acs.jcim.5c00354

**Published:** 2025-07-28

**Authors:** Karl Thurnhofer-Hemsi, Iván García-Aguilar, José David Fernández-Rodriguez, Ezequiel López-Rubio

**Affiliations:** † ITIS Software, Universidad de Málaga, C/Arquitecto Francisco Peñalosa 18, Málaga 29010, Spain; ‡ Biomedic Research Institute of Málaga, IBIMA Plataforma BIONAND, C/Doctor Miguel Díaz Recio, 28, Málaga 29010, Spain

## Abstract

The processing of chemical information by computational intelligence methods faces the challenge of the structural complexity of molecular graphs. These graphs are not amenable to being represented in a suitable way for such methods. The most popular representation is the SMILES notation standard. However, it comes with some limitations, such as the abundance of nonvalid strings and the fact that similar strings often represent very different molecules. In this work, a completely different approach to chemical nomenclature is presented. A reduced instruction set is defined, and the language of all strings that are sequences of such instructions is considered. Instructions provide the means to incrementally add atoms and modify the connectivity of the chemical bonds of atoms to be inserted. Instructions are carefully crafted to guarantee that all strings of this language are valid, i.e., each string represents a molecule. Moreover, slight changes in a string usually correspond to small modifications in the represented molecule. Therefore, this approach is appropriate for use in state-of-the-art computational intelligence systems for chemical information processing, including deep learning models.

## Graphical abstract

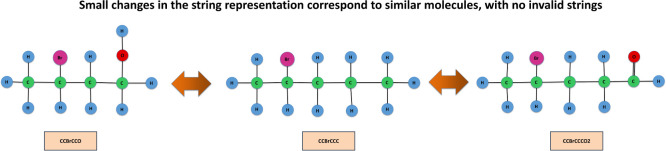

## Introduction

The SMILES (Simplified Molecular Input Line Entry System) notation^1,2^ offers an efficient method for encoding the structure of chemical molecules in strings of characters. This approach simplifies the computational processing of molecular structures and has become a standard in the industry.

The description of graph structures, such as chemical compounds, by means of strings of characters is typically done by the definition of a description language, which, from the point of view of computer science, is a context-free language. This means that pairs of matched tokens in the string, such as parentheses, are used to denote the branches and cycles in the molecular structure. This strategy follows from the traditional chemical notation, which also employs pairs of parentheses. In particular, the standard SMILES notation linearizes chemical graphs by pairs of parentheses and pairs of matching numbered tokens. Nesting and stacking of these syntactic structures in the strings allow the representation of any molecule.

However, context-free languages pose significant difficulties for computational intelligence processing of chemical information. Any mismatch in the paired tokens inevitably leads to an invalid string, i.e., a string that does not represent any molecule because it does not conform to the syntax rules of the context-free language. Critically, optimization of molecular structures becomes challenging because it is not straightforward to make a small change in a valid string to obtain another valid string that represents a similar molecule. Furthermore, the use of deep learning generative neural models is hindered by the generation of nonvalid strings, as well as the sharp changes in the chemical properties that a small change in a string may produce. The most established way to evaluate such chemical similarity consists of employing suitable molecular fingerprints,^3^ followed by the evaluation of fingerprint-based similarity metrics.

This paper presents an approach to the representation of chemical compounds that aims to overcome these shortcomings. The proposed strategy is based on an instruction set that can be used to build a molecule step by step, in a sequential way. The associated language of possible strings is very simple, and since it is a regular language, it is more suitable for direct processing by computational intelligence algorithms and methods.

The structure of this paper is as follows. First, some works related to our investigation are reviewed. Then, the proposed molecule representation methodology is detailed. After that, some illustrative examples are provided, and the key properties of the proposal are considered. Computational experiments and discussion follow. Finally, conclusions and future works are given.

## Background

There are many line notations for chemical structures, and some of them date to the 1950s.^4^ Other digital ways of storing molecular information, such as MDL molfile^5^ are not relevant to this work because they are computer file formats and not line notations, so they will not be reviewed here. However, they are essential for chemical database construction and management.^6–8^ Also, other chemical identifiers such as CAS RN will not be discussed either because they do not convey the structural information on the molecule explicitly, but they are keys to query molecular databases.^9,10^

The SMILES line notation is a worldwide standard together with the IUPAC International Chemical Identifier^11–13^ (InChI). Both are based on depth-first traversal of the molecular graph to be represented to yield a string representation. InChI is a notation that provides several layers of detail in the description of a molecule and is based on the connection table of the molecule. The atoms in the molecule are given a number depending on their position in the chain of connected atoms. Each atom is referred to in the string by its number. For computational intelligence purposes, this has three main disadvantages. First of all, strings that make reference to nonexistent atom numbers are invalid. Second, any insertion or deletion in the chain leads to a nearly complete change in the atom numbers. Third, the identifying numbers of some atoms that exist in the molecule may not appear in the string, which again leads to an invalid string. Therefore, the chances that a small change in an InChI string leads to an invalid one are high. In other words, the situation is worse than for SMILES.

The comparatively good characteristics of SMILES for the generation and optimization of molecular structures have driven it to be the notation of choice in order to develop deep learning models that manage molecules.^14,15^ Nevertheless, InChI or binary fingerprints are sometimes employed.^16^ It is even possible to provide two different representations to the same deep network, for example, SMILES and InChI.^17^ SMILES strings are often required to be mapped to numeric vectors prior to processing by deep learning neural networks.^18^ In view of the deleterious effect of invalid SMILES strings, the SELFIES notation has been introduced^19^ which has no invalid strings. However, SELFIES does not address another key issue, namely the requirement that similar strings represent molecules with a high chemical similarity. This property is needed in order to facilitate the progressive learning and optimization processes that operate within computational intelligence models. In other words, connectivity (topology) among valid strings that is meaningful from the chemical point of view is required (i.e., given similar strings, they should map to similar chemical structures). Interestingly enough, it has been reported that deep learning models based on SMILES tokenization exhibit better performance than those based on SELFIES.^20^

Many inconsistencies have been reported within and between molecule databases.^21^ That is, the representations of a molecule (molfiles, SMILES strings, IUPAC names, and InChI strings) do not match. This inconvenience can be alleviated by having a normalized version of each notation. Hence, a secondary goal of any new proposal for molecular notation is to define a normalized form. In this respect, the InChI notation is somehow better than SMILES because InChI guarantees a unique representation of each molecule^15^ with some rare exceptions.^22^ It has been proposed to employ InChI to guide the canonicalization of SMILES strings.^23^ Another approach is to apply a molecular graph canonicalization algorithm, typically the Morgan algorithm, although there are other proposals.^24^ Standardization of chemical structures is easier to accomplish whenever such normalized versions are available.^25^

## Methodology

In this section, the proposal for the representation of chemical compounds is detailed. The strategy is based on an instruction set so that a molecule is represented by a sequence of instructions from the set. In order to obtain the graph of the molecule, the instructions in the string must be executed sequentially, from left to right. That is, each string is interpreted as a program that specifies how to build a molecular graph from scratch. The set of such strings forms the language of all possible representations under this notation.

### Instruction Set and String Execution

The defined instructions are as simple as possible, in order to facilitate the interpretation of the strings and also to reduce the differences among the structures of the molecules represented by similar strings. An analogy can be drawn between these strings and computer machine code, which is made of sequences of machine language instructions.

The execution of a string to produce the graph of a molecule proceeds as follows. From an initial state, the string is scanned from left to right. Each token in the string is an instruction to be executed that transforms the current state into the next state. That is, each instruction operates on the current state to produce the next state. When the string is exhausted, the execution halts, and the molecular graph contained in the current state is yielded as the result, i.e., it is the molecule that is represented by the string.

Each possible state contains four data structures:

- The molecular graph. Each node in the graph represents an atom. Each edge in the graph represents a bond (simple, double, or triple) between two atoms. The outgoing bonds of an atom are ordered; for example, the four bonds of a carbon atom are numbered as 0, 1, 2, and 3.
- The list of hydrogen atoms. This is a list that enumerates all the nodes in the graph that represent hydrogen atoms. The list is circular, i.e., the first node’s previous node is the last node, and the last node’s next node is the first node.
- The primary pointer. It is a pointer to one of the nodes in the graph that represents a hydrogen atom.
- The secondary pointer. It is another pointer to one of the nodes in the graph that represents a hydrogen atom. Both pointers may point to the same node.

The initial state contains a graph with just two nodes and an edge connecting them, which represents molecular hydrogen, i.e., two hydrogen atoms linked by a single bond. The list of hydrogen atoms only contains two nodes. Finally, the primary pointer points to one of the nodes, and the secondary pointer points to the other node in the graph.

The instruction set is defined as follows. First of all, the subset of instructions that do not modify the graph is given; the token associated with each instruction is given in parentheses:

- No Operation (A). No change is performed in the current state.
- Increase primary pointer (+). The primary pointer is moved to the next node in the list of hydrogen atoms.
- Decrease primary pointer (−). The primary pointer is moved to the previous node in the list of hydrogen atoms.
- Increase secondary pointer (>). The secondary pointer is moved to the next node in the list of hydrogen atoms.
- Decrease secondary pointer (<). The secondary pointer is moved to the previous node in the list of hydrogen atoms.

Second, the subset of instructions that modify the graph by adding single bonds is as follows:

- Insert single-bond carbon (C). The hydrogen atom pointed by the primary pointer is removed and substituted by a carbon atom. The new carbon atom is linked to the graph by its bond number 0 at the location of the old hydrogen atom. Three new hydrogen atoms are inserted with bonds to the new carbon atom at bond numbers 1, 2, and 3. The old hydrogen atom is removed from the list, and the three new hydrogen atoms are inserted at that position in the list, sorted by their bond numbers. The primary pointer is updated to point to the second new hydrogen atom, i.e., the new hydrogen atom that is linked to bond number 2 of the new carbon atom. If the secondary pointer was pointing to the old hydrogen atom, then it is updated to also point to the second new hydrogen atom.
- Insert single-bond nitrogen/boron (N/B). The hydrogen atom pointed by the primary pointer is removed and substituted by a nitrogen/boron atom. Two hydrogen atoms are inserted with bonds to the new nitrogen/boron atom. The old hydrogen atom is removed from the list, and the two new hydrogen atoms are inserted at that position in the list. The primary pointer is updated to point to the second new hydrogen atom. If the secondary pointer was pointing to the old hydrogen atom, then it is updated to also point to the second new hydrogen atom.
- Insert single-bond oxygen (O). The hydrogen atom pointed by the primary pointer is removed and substituted by an oxygen atom. One hydrogen atom is inserted with a bond to the new oxygen atom. The old hydrogen atom is removed from the list, and the new hydrogen atom is inserted at that position in the list. The primary pointer is updated to point to the new hydrogen atom. If the secondary pointer was pointing to the old hydrogen atom, then it is updated to also point to the new hydrogen atom.
- Insert halogen (F/Cl/Br/I). The hydrogen atom pointed by the primary pointer is removed and substituted by a halogen atom. The old hydrogen atom is removed from the list. The primary pointer is updated to point to the previous hydrogen atom in the list. If the secondary pointer was pointing to the old hydrogen atom, then it is updated to also point to the previous hydrogen atom in the list.

Then, the subset of instructions that modify the graph by adding a double bond is considered. They include provisions to fall back to their respective single-bond versions in case there are not enough hydrogen atoms:

- Insert double-bond carbon (C2). If the list only contains one hydrogen atom, or the hydrogen atom pointed by the primary pointer and the next hydrogen atom in the list are not bound to the same non-hydrogen atom, then instruction C is executed. Otherwise, the hydrogen atom pointed by the primary pointer and the next hydrogen atom in the list are removed and substituted by a carbon atom. The new carbon atom is linked to the graph by its bonds 0 and 1 at the location of the two old hydrogen atoms. Two hydrogen atoms are inserted with bonds to the new carbon atom at bond numbers 2 and 3. The old hydrogen atoms are removed from the list, and the two new hydrogen atoms are inserted at that position in the list. The primary pointer is updated to point to the second new hydrogen atom. If the secondary pointer was pointing to any of the two old hydrogen atoms, then it is updated to also point to the second new hydrogen atom.
- Insert double-bond nitrogen/boron (N2/B2).If the list only contains one hydrogen atom, or the hydrogen atom pointed by the primary pointer and the next hydrogen atom in the list are not bound to the same non-hydrogen atom, then instruction N/B is executed. Otherwise, the hydrogen atom pointed by the primary pointer and the next hydrogen atom in the list are removed and substituted by a nitrogen/boron atom. A hydrogen atom is inserted with a bond to the new nitrogen/boron atom. The old hydrogen atoms are removed from the list, and the new hydrogen atom is inserted at that position in the list. The primary pointer is updated to point to the new hydrogen atom. If the secondary pointer was pointing to any of the two old hydrogen atoms, then it is updated to also point to the new hydrogen atom.
- Insert double-bond oxygen (O2). If the list only contains one hydrogen atom, or the hydrogen atom pointed by the primary pointer and the next hydrogen atom in the list are not bound to the same non-hydrogen atom, then instruction O is executed. Otherwise, the hydrogen atom pointed by the primary pointer and the next hydrogen atom in the list are removed and substituted by an oxygen atom. The old hydrogen atoms are removed from the list. The primary pointer is updated to point to the previous hydrogen atom in the list. If the secondary pointer was pointing to any of the two old hydrogen atoms, then it is updated to also point to the previous hydrogen atom in the list.

After that, the subset of instructions that modify the graph by adding a triple bond is given. They fall back to their respective double-bond versions in case there are not enough hydrogen atoms:

- Insert triple-bond carbon (C3). If the list contains less than three hydrogen atoms, or the hydrogen atom pointed by the primary pointer and the previous and next hydrogen atoms in the list are not bound to the same non-hydrogen atom, then instruction C2 is executed. Otherwise, the hydrogen atom pointed by the primary pointer and the previous and next hydrogen atoms in the list are removed and substituted by a carbon atom. The new carbon atom is linked to the graph by its bonds 0, 1, and 2 at the location of the three old hydrogen atoms. One hydrogen atom is inserted with a bond to the new carbon atom at bond number 3. The old hydrogen atoms are removed from the list, and the new hydrogen atom is inserted at that position in the list. The primary pointer is updated to point to the new hydrogen atom. If the secondary pointer was pointing to any of the three old hydrogen atoms, then it is updated to also point to the new hydrogen atom.
- Insert triple-bond nitrogen/boron (N3/B3). If the list contains less than three hydrogen atoms, or the hydrogen atom pointed by the primary pointer and the previous and next hydrogen atoms in the list are not bound to the same non-hydrogen atom, then instruction N2/B2 is executed. Otherwise, the hydrogen atom pointed by the primary pointer and the previous and next hydrogen atoms in the list are removed and substituted by a nitrogen/boron atom The old hydrogen atoms are removed from the list. The primary pointer is updated to point to the previous hydrogen atom in the list. If the secondary pointer was pointing to any of the three old hydrogen atoms, then it is updated to also point to the previous hydrogen atom in the list.

Finally, instructions are defined that manage cyclic molecular structures. Again, a fallback is defined in case a double bond cannot be inserted:

- Insert single-bond joint (J). If the hydrogen atoms pointed by the primary and secondary pointers are bound to the same non-hydrogen atom, then the instruction is ignored, i.e., no operation is performed. Otherwise, the hydrogen atoms pointed by the primary and secondary pointers are removed and substituted by a single bond. Both old hydrogen atoms are removed from the list. The primary pointer is updated to point to the previous remaining hydrogen atom in the list. Similarly, the secondary pointer is updated to point to the previous remaining hydrogen atom in the list.
- Insert double-bond joint (J2). If the hydrogen atoms pointed by the primary and secondary pointers are bound to the same non-hydrogen atom, then the instruction is ignored, i.e., no operation is performed. Next, if the hydrogen atom pointed by the primary pointer and the next hydrogen atom in the list are not bound to the same non-hydrogen atom, or the hydrogen atom pointed by the secondary pointer and the next hydrogen atom in the list are not bound to the same non-hydrogen atom, then instruction J is executed. Otherwise, the hydrogen atoms pointed to by the primary and secondary pointers and their respective successors in the list are removed and substituted by a double bond. The four old hydrogen atoms are removed from the list. The primary pointer is updated to point to the previous remaining hydrogen atom in the list. Similarly, the secondary pointer is updated to point to the previous remaining hydrogen atom in the list.

Table 1 summarizes the above-defined IsalChem instruction set. Please note that specific instructions to insert aromatic structures are not necessary since aromaticity can be automatically detected and annotated after the molecular graph is generated. There is no need for an instruction to insert a triple-bond joint, either, because there cannot be a cycle composed of triple bonds only.
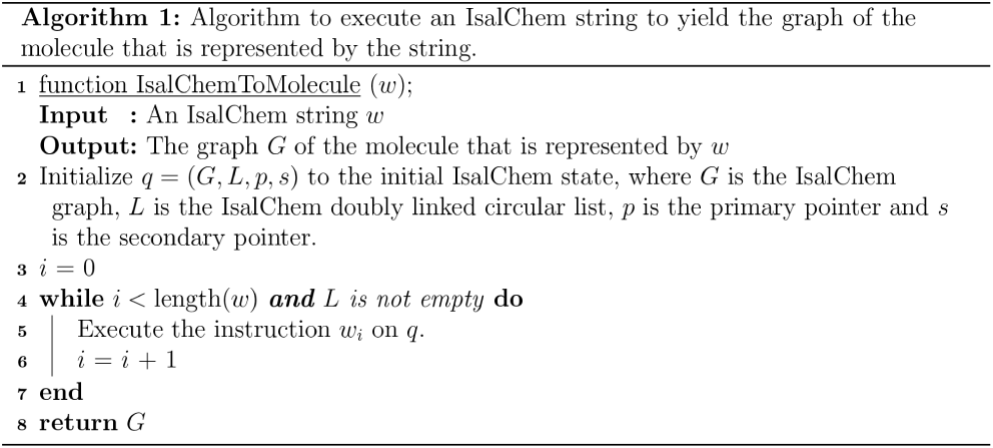


**Table 1:** IsalChem Instruction Set

Instruction group	Instruction	Description
No operation	A	No operation
Pointer movement	+	Increase primary pointer
	–	Decrease primary pointer
	>	Increase secondary pointer
	<	Decrease secondary pointer
Single bond insertion	C	Insert single-bond carbon
	N/B	Insert single-bond nitrogen/boron
	O	Insert single-bond oxygen
	F/Cl/Br/I	Insert halogen
Double bond insertion	C2	Insert double-bond carbon
	N2/B2	Insert double-bond nitrogen/boron
	O2	Insert double-bond oxygen
Triple bond insertion	C3	Insert triple-bond carbon
	N3/B3	Insert triple-bond nitrogen/boron
Cycle insertion	J	Insert single-bond joint
	J2	Insert double-bond joint

If the list of hydrogen atoms becomes empty after performing an instruction, then the execution halts, and the molecular graph contained in the current state is given as the final result.

The above specification can be employed to represent chemical compounds in two ways:

- Variable length strings. Only the minimum required instructions are given in the string.
- Fixed-length strings. A fixed length is established for the strings beforehand. Then, the required instructions are given in the string, with extra leading and trailing no-operation instructions (A) as needed to complete the established string length.

It must be highlighted that the position where no-operation instructions are added in a string is irrelevant, i.e., the represented molecule is the same. It is also irrelevant how many no-operation instructions are added. A color key for the subsequent figures in this work is provided in Figure 1.

**Figure 1:**
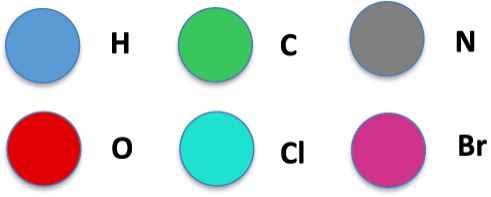
Color key for the chemical elements.

For stereochemistry purposes, it is necessary to specify how the numbering of the four bonds of a carbon atom is rendered in the three-dimensional space. This way, the orientation of the four bonds of a chiral center is defined. If the carbon atom is oriented in space so that bond number 0 points away from the observer, then bonds 1, 2, and 3 form a triangle where the three vertices are numbered counterclockwise, as depicted in Figure 2. That is, a sinister (S) assignment is defined.

**Figure 2:**
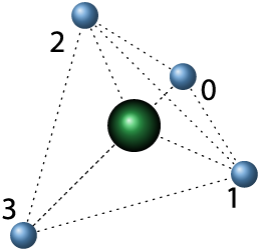
Rendering of the bonds of a carbon atom for a chiral center. The green ball is the carbon atom. The four blue balls are hydrogen atoms. The atom number 0 points away from the observer.

Also, it is necessary to specify the geometric arrangement of the bonds of two carbon atoms connected by a double bond. This way, *cis–trans* isomerism can be managed. As established in the definition of the C2 and J2 instructions above, the two bonds that comprise a double bond must have consecutive numbers. Let us note that *x* and *x*+1 are the numbers associated with the double bond in the first carbon atom, where *x* ∈ {0,1,2}. Also, let us note that *y* and *y*+1 are the numbers associated with the double bond in the second carbon atom, where *y* ∈ {0,1,2}. Then, the bond numbers for the bonds that are next to and previous to the double bond in the first carbon atom are noted *a* and *b*, respectively:

1 a=(x+2)mod⁡4

2 b=(x−1)mod⁡4

where mod stands for the remainder of the integer division.

Furthermore, the bond numbers for the bonds that are next and previous to the double bond in the second carbon atom are noted *c* and *d*, respectively, where

3 c=(y+2)mod⁡4

4 d=(y−1)mod⁡4

The geometric arrangement is depicted in Figure 3. If the substituents are in bonds *a* and *d*, or *b* and *c*, then the configuration is *cis*, i.e., the substituents are on the same side. If the substituents are in bonds *a* and *c*, or *b* and *d*, then the configuration is *trans*, i.e., the substituents are on opposite sides.

**Figure 3:**
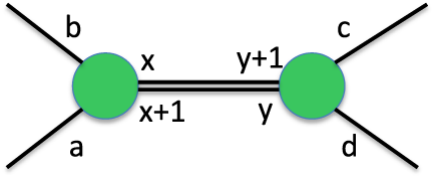
Arrangement of the bonds of a double bond between two carbon atoms for *cis–trans* isomerism. The green circles are the carbon atoms.

The above-presented methodology is called IsalChem, which stands for Instruction Set And Language for CHEMical nomenclature. The strings that follow this nomenclature are called IsalChem strings, and the set of all such strings is the IsalChem language. Algorithm 1 summarizes the procedure to obtain the graph of the molecule that is represented by an IsalChem string. An IsalChem state *q* = (*G*,*L*,*p*,*s*) is maintained by the algorithm, where *G* is the IsalChem graph, *L* is the IsalChem doubly linked circular list, *p* is the primary pointer and *s* is the secondary pointer. Please note that zero-based string indices are used, i.e., the first instruction of string *w* is *w*_0_.

### Molecule to String Conversion

In order to employ the IsalChem methodology in a practical setting, a conversion algorithm is required to automatically convert a molecular graph into an IsalChem string. A proposal for this task is detailed next.

Algorithm 2 takes as input the molecular graph *M* to be converted into an IsalChem string. An IsalChem state *q* = (*G*,*L*,*p*,*s*) is iteratively updated on each iteration of its main loop. In addition to this, a set *R* of atoms from the input molecular graph *M* is also maintained, which contains the non-hydrogen atoms that are waiting to be connected to an atom of the output IsalChem graph *G*. The non-hydrogen atoms in *R* are bound in *M* to at least one non-hydrogen atom in *G*. The IsalChem string is iteratively built as *w*, and the current state *q* is updated every time that an instruction is appended to *w*. The temporary graph *G* is grown until it matches the input graph *M*. When the set of waiting non-hydrogen atoms *R* becomes empty, the conversion is finished.

Movements of the primary pointer are minimized by searching for the non-hydrogen atom waiting to be inserted into *G* that is closest to the current location of the primary pointer. This way, the length of the produced string *w* is reduced.

### String Compression and Normalization

Any molecule has many IsalChem strings that represent it, and some of which may be very long. This is because no-operation instructions can be added freely, and also because pointer movement instructions may cancel each other if there are no atom insertion instructions among them. This calls for a string compression algorithm that removes all these redundancies and yields a short string. As an additional benefit, the output of this algorithm may be employed as the normalized (canonical) IsalChem string in case one must be defined for a particular task.

Algorithm 3 is the string compression procedure. It consists of the repeated application of the conversion algorithm from the molecular graph to a string (Algorithm 2), where a different way to visit the nodes of the graph is explored on each iteration. Please note that among all strings of the shortest length, length­(*y*) < length­(*z*), the string that comes first in lexicographical order (*y* < *z*) is chosen as the final output. This yields a normalized (canonical) string to represent a given molecule. Normalization can be employed for all purposes where a unique string representation of a molecule is required.

The reproducibility and uniqueness of normalized IsalChem strings are studied next. All IsalChem strings are reproducible, not only the standardized ones, because the conversion from an IsalChem string to a molecular graph (Algorithm 1) is fully detailed and deterministic[Fn ba-fn1].

Regarding uniqueness, the normalization algorithm (Algorithm 3) starts by converting the input string into its associated molecular graph. The obtained molecular graph may be assumed to be unique for a given molecular species. From this point, the normalization algorithm uses the molecular graph to produce the normalized string. This process is not affected by graph isomorphism, i.e., renaming of the atom labels, because Algorithm 3 does not depend on the specific contents of the atom labels. Nevertheless, there are several ways that Algorithm 2, which is called by Algorithm 3, can visit the nodes (atoms) of the graph. These ways come from the nondeterministic selections at steps 7 and 9 of Algorithm 2. This nondeterminism is managed in Algorithm 3 by exploring all possible options in steps 7 and 9 of Algorithm 2. Algorithm 3 has been updated to explore all possibilities explicitly. This way, it is ensured that the normalized IsalChem string is unique.
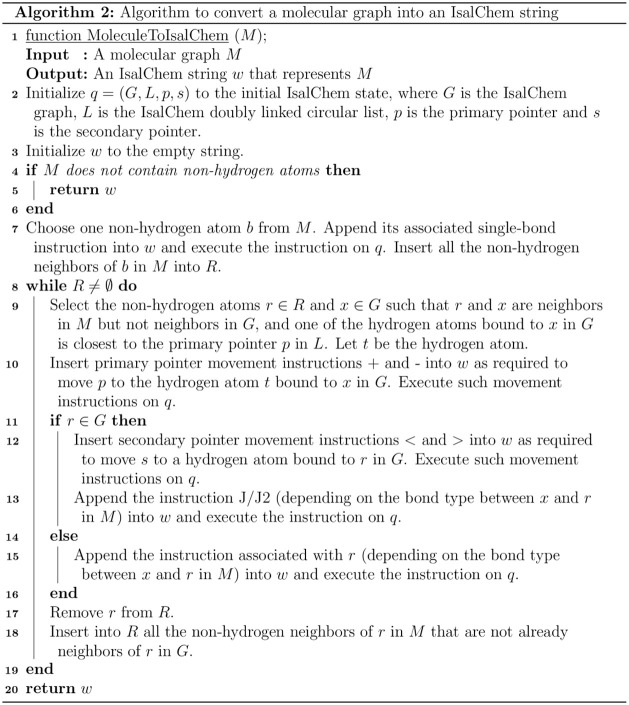


If computational complexity must be reduced, then it suffices to execute just one iteration of the loop in order to remove most redundancies in the input string. As an intermediate option in terms of computational load, a random subset of the non-hydrogen atoms of the molecular graph can be tried as initializations for Algorithm 2.
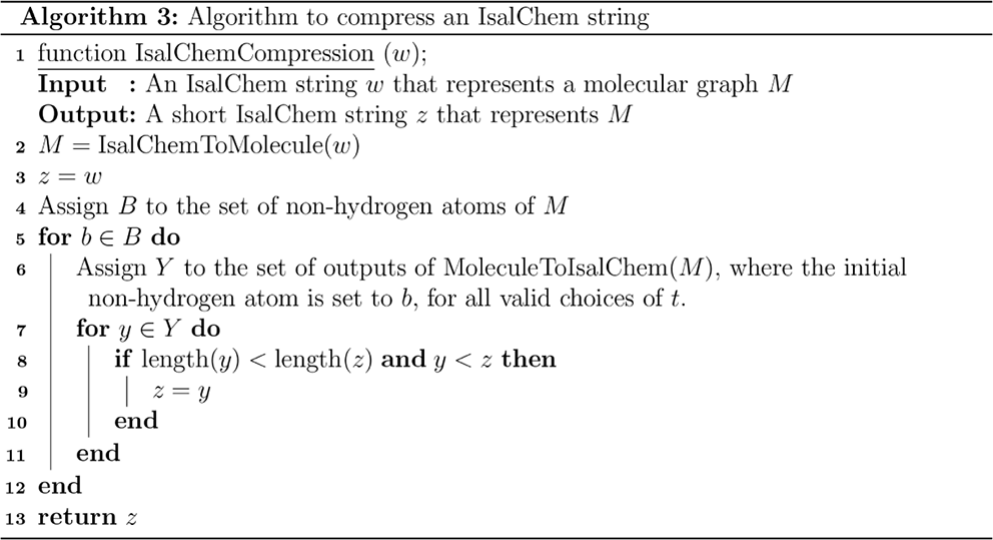


## Examples

In this section, several examples are provided to showcase the proposal. In the included figures that depict IsamChem states, atoms are shown as circles of different colors depending on the chemical element (see the color key in Figure 1), chemical bonds are marked with solid lines, hydrogen atom list connections are marked with dashed lines, the atom pointed by the primary pointer is surrounded by a red square, and the atom pointed by the secondary pointer is surrounded by a blue square. Each atom is labeled with the index of the token in the IsalChem string that gave rise to the atom, starting with index zero. Please note that the two initial hydrogen atoms are not labeled. Also, please note that the indices refer to the tokens and not to the characters, i.e., a token may comprise one (for example, O) or two (for example, N3) characters.

First of all, the execution of a string is given in order to visualize the operation of the defined instruction set. Figure 4 shows the sequence of IsalChem states that are produced by the execution of the string CCO2+O, which represents hydroxyacetaldehyde. As shown in the Figure, the non-hydrogen atoms are inserted one by one and sequentially at the primary pointer location, according to the instructions in the string, and hydrogen atoms are added or removed adequately. Another example is available in the Supporting Information.

**Figure 4:**
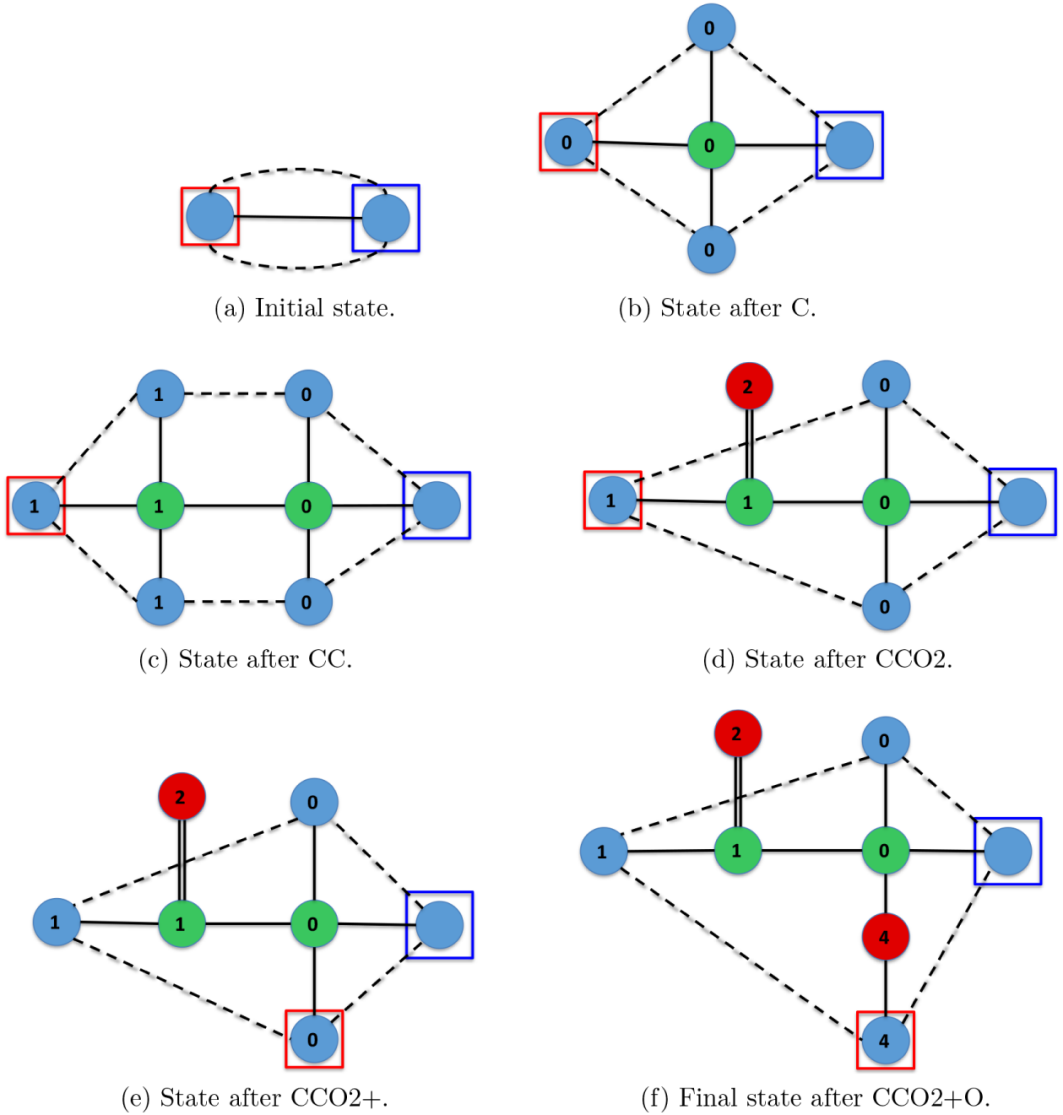
Execution of the string CCO2+O, corresponding to hydroxyacetaldehyde.

Next, a set of six molecules has been chosen to provide additional examples of the proposal. Table 2 lists six example molecules along with their string representations in the SMILES and IsalChem notations. The number of tokens of each representation is also provided. Please note that each instruction is one token in IsalChem. As seen, both notations exhibit a similar number of tokens, with IsalChem using a slightly smaller number of tokens in some cases.

**Table 2:** Examples of Representations

Molecule	System	Representation	Tokens
1,4-hexadiene	SMILES	C(C=C)C=CC	10
	IsalChem	CCC2CCC2	6
3-propyl 4-isopropyl 1-heptene	SMILES	C=CC(CCC)C(C(C)C)CCC	20
	IsalChem	CC2CCCCC++++CC++C++CCC	21
Cyclohexane	SMILES	C1CCCCC1	8
	IsalChem	CCCCCCJ	7
Isobutyric acid	SMILES	CC(C)C(=O)O	11
	IsalChem	CCC–O+O2+C	9
Triethylamine	SMILES	CCN(CC)CC	9
	IsalChem	CCNCC---CC	10
2,4,5-trichlorophenol	SMILES	Clc1cc(O)c(Cl)cc1Cl	16
	IsalChem	CC2CC2CC2JO–Cl–ClCl	13
Flavone	SMILES	C1=CC=C(C=C1) C2=CC(=O)C3=C C003dCC=C3O2	35
	IsalChem	CCC2CC2CJ2++>++CO2CC2OJ≫C<C2CC2CC2J	27

Furthermore, Figures 5–11 depict the final states associated with the execution of the IsalChem strings provided in Table 2. It is remarkable that even for molecules with a more complex structure, the list of hydrogen atoms remains pretty untangled, i.e., most links between pairs of hydrogen atoms in the list correspond to atoms that are bound to the same non-hydrogen atom or a nearby non-hydrogen atom (except for the more complex case of flavone in Figure 11, whose links are slightly entangled). This is due to the design of the atom insertion instructions. Consequently, the pointer modification instructions (+, −, >, and <) move the primary and secondary pointers smoothly across the molecular structure. This demonstrates that the proposal preserves the locality of the graph modifications carried out by the instructions.

**Figure 5:**
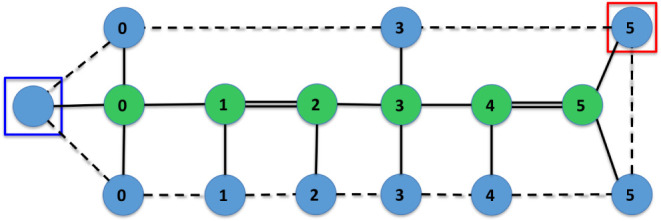
Diagram of the final state for the execution of the CCC2CCC2 string, corresponding to the 1,4-hexadiene molecule.

**6:**
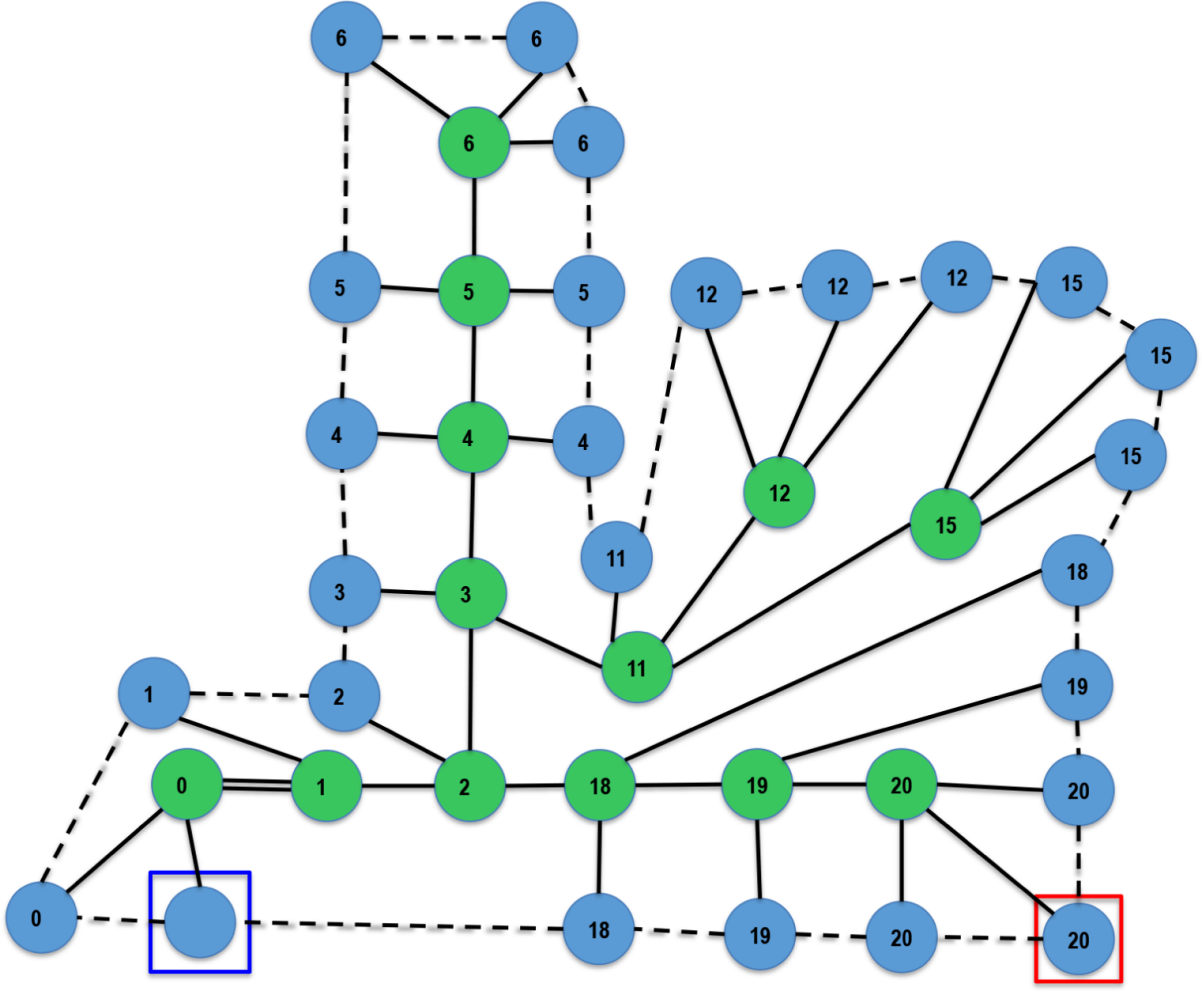
Diagram of the final state for the execution of the CC2CCCCC++++CC++C++CCC string, corresponding to the 3-propyl 4-isopropyl 1-heptene molecule.

**7:**
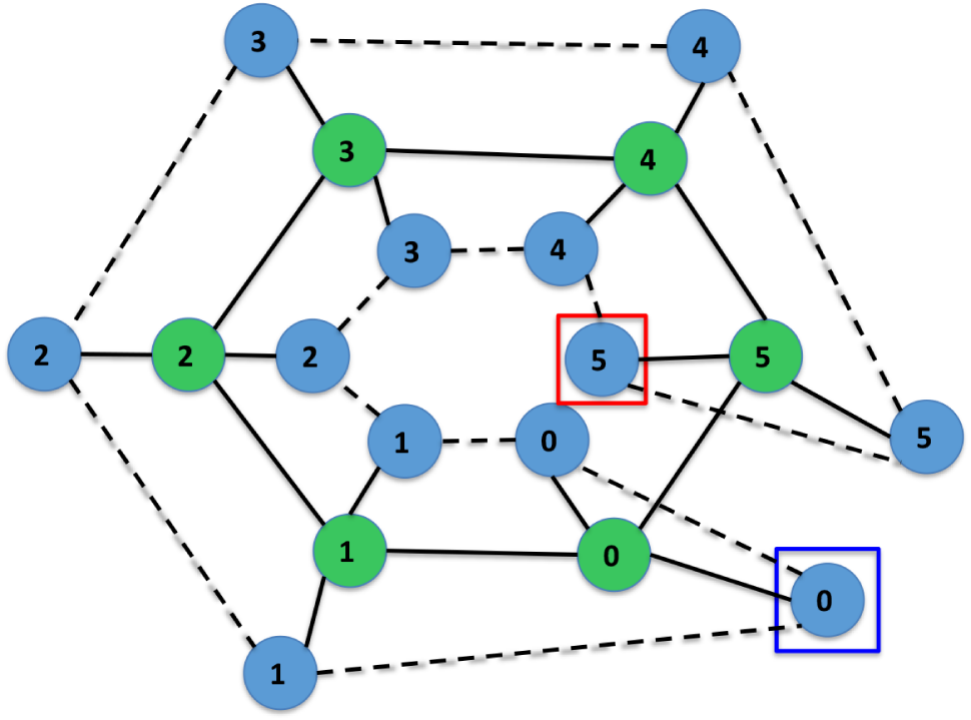
Diagram of the final state for the execution of the CCCCCCJ string, corresponding to the cyclohexane molecule.

**8:**
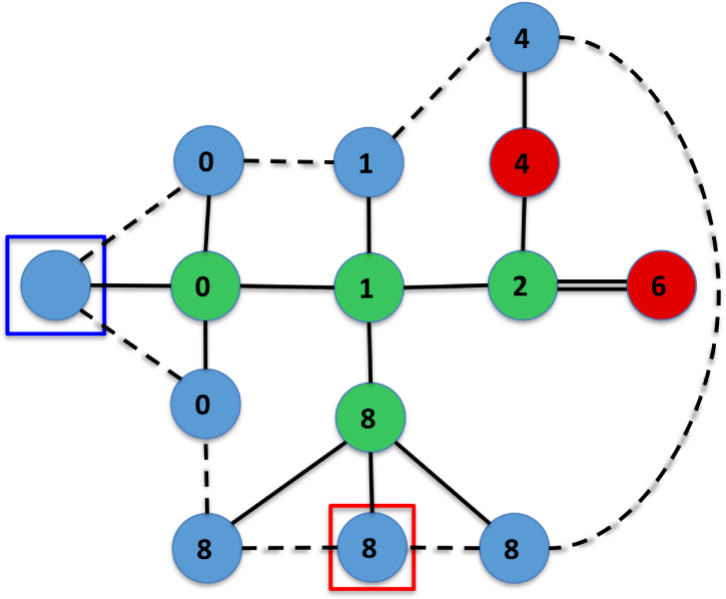
Diagram of the final state for the execution of the CCC-O+O2+C string, corresponding to the isobutyric acid molecule.

**9:**
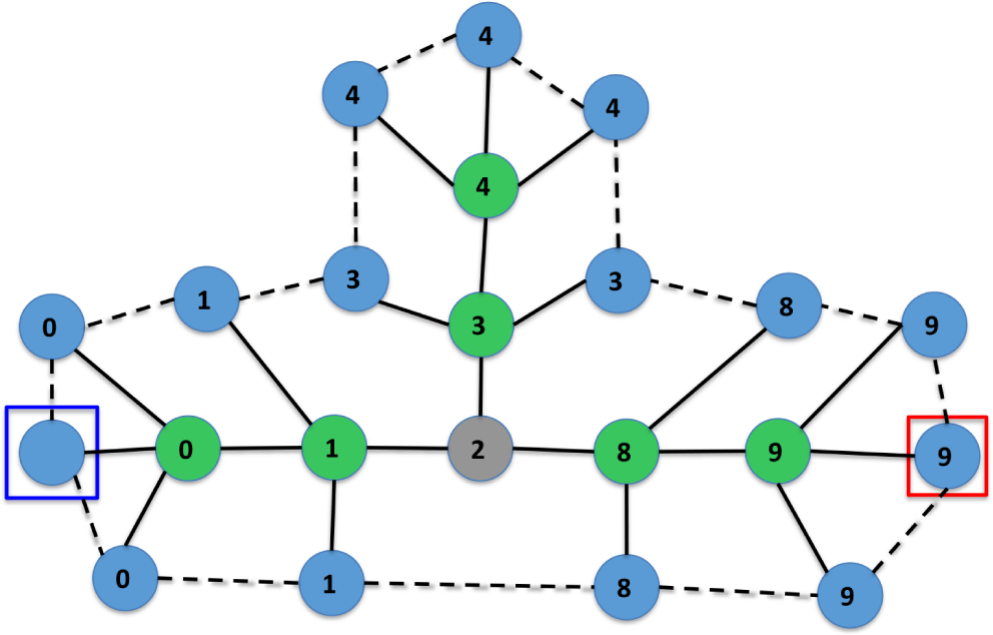
Diagram of the final state for the execution of the CCNCC---CC string, corresponding to the triethylamine molecule.

**10:**
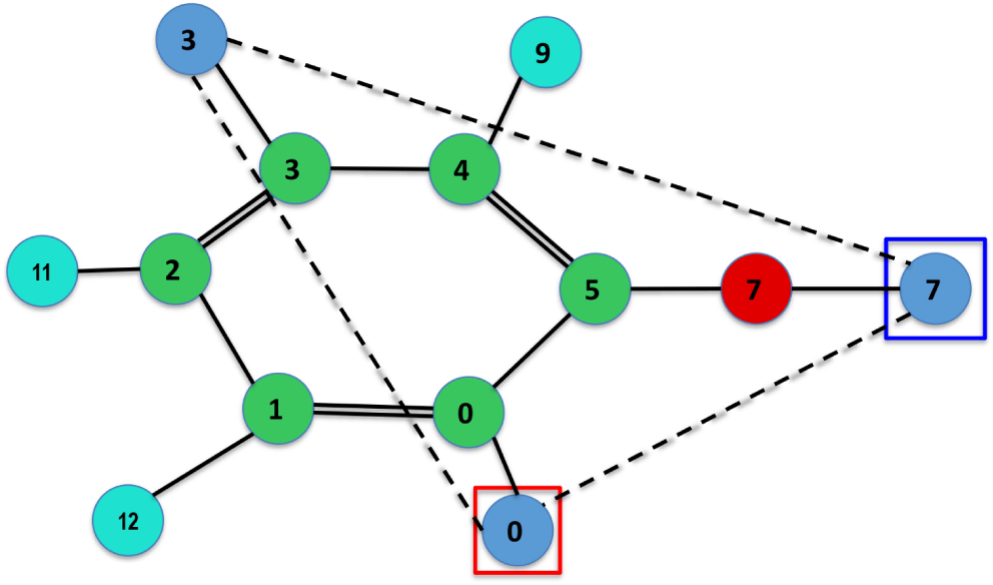
Diagram of the final state for the execution of the CC2CC2CC2JO–Cl–ClCl string, corresponding to the 2,4,5-trichlorophenol molecule.

**Figure 11:**
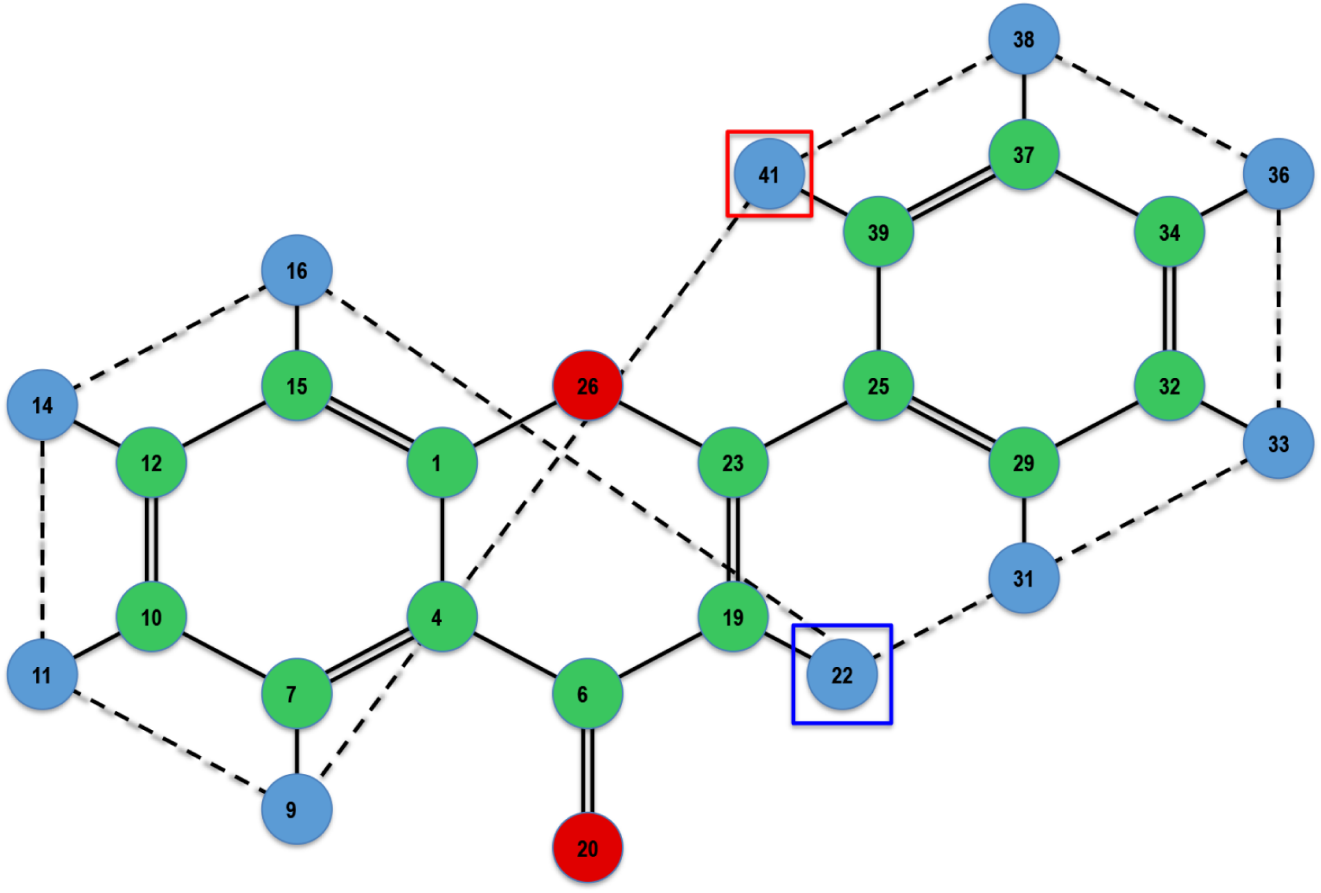
Diagram of the final state for the execution of the string corresponding to flavone (the string is shown in the last entry in Table 2).

Figure 11 depicts flavone, a slightly more complex example than the ones in previous Figures. Flavone has three rings, two of them fused. The string proposed to build flavone can be divided into three concatenated substrings:

- CCC2CC2CJ2: The first substring builds the ring depicted to the left in Figure 11.
- ++>++CO2CC2OJ: The second substring builds the central ring, fused to the previous one, i.e., sharing two carbons with it. To build this fused ring, the primary and secondary pointers are positioned in each of the two shared carbons; then the central ring is built.
- ≫C<C2CC2CC2J: The third substring builds the last ring, linked to the central one by a bond with one carbon. To build this ring, the primary and secondary pointers are positioned in the central ring’s carbon that is to be bonded to the last ring. Finally, the last ring is built.

Next, some examples of stereochemistry are provided. On one hand, Figures 12 and 13 depict two isomers, *R*-(−)-2-butanol and (S)-(+)-2-butanol, respectively. They differ in the configuration of a chiral center. As seen in the figures, the change of a pointer movement instruction from + to – specifies the difference between the two isomers. This is because the substituents at the chiral center are positioned at different bond numbers due to this change. On the other hand, Figures 14 and 15 represent a pair of *cis–trans* isomers, namely *cis*-but-2-ene and *trans*-but-2-ene, respectively. In this case, the absence or presence of the – pointer movement instruction switches between the *cis* and *trans* configurations. Again, this is because the substituents are positioned at different bond numbers depending on the presence of the – pointer movement instruction.

**Figure 12:**
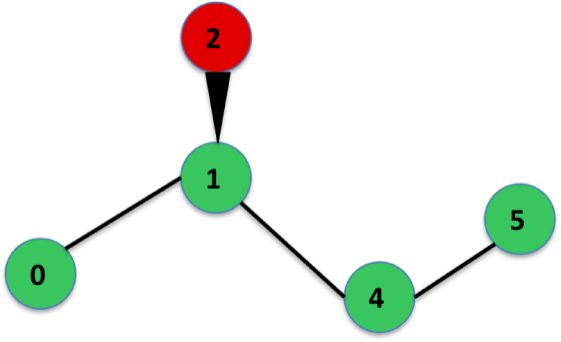
Diagram of the final state for the execution of the CCO+CC string, corresponding to the *R*-(−)-2-butanol molecule. The hydrogen atoms have been omitted for the sake of simplicity. Oxygen atom number 2 is pointing toward the observer.

**Figure 13:**
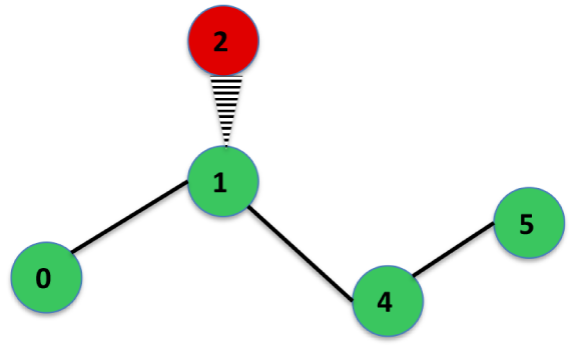
Diagram of the final state for the execution of the CCO–CC string, corresponding to the (S)-(+)-2-butanol molecule. The hydrogen atoms have been omitted for the sake of simplicity. Oxygen atom number 2 is pointing away from the observer.

**Figure 14:**
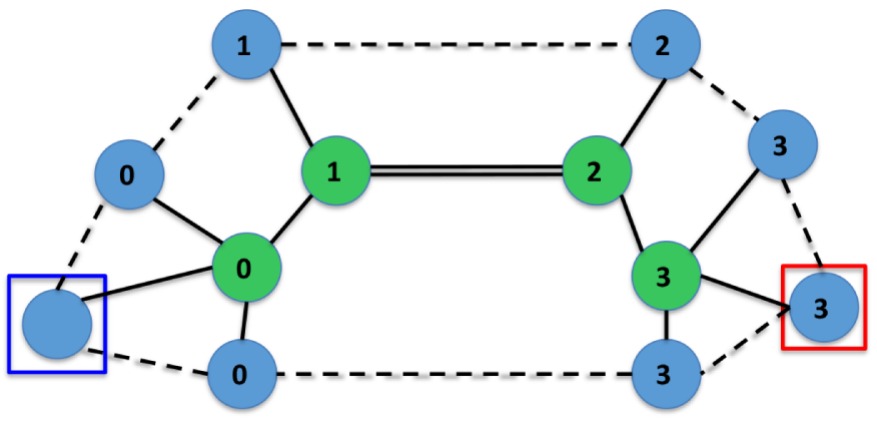
Diagram of the final state for the execution of the CCC2C string, corresponding to the cis-but-2-ene molecule.

**Figure 15:**
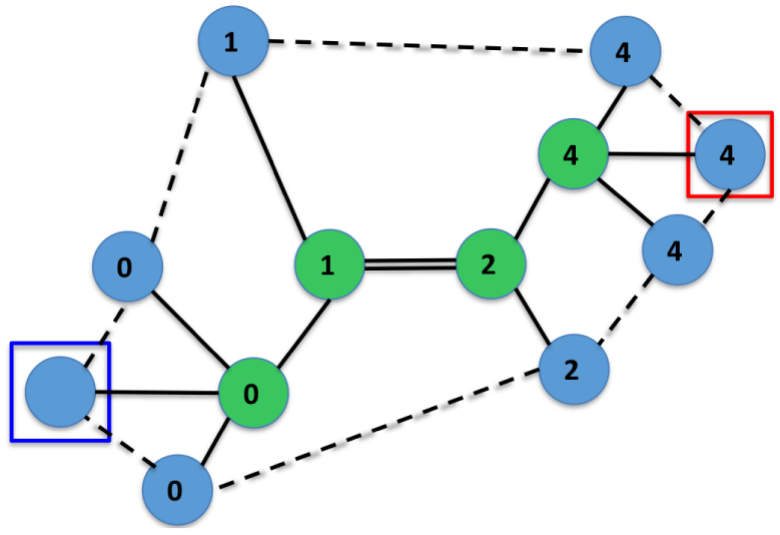
Diagram of the final state for the execution of the CCC2-C string, corresponding to the trans-but-2-ene molecule.

Finally, Figures 16 and 17 depict a trace of the execution of Algorithm 3, which converts a molecular graph *M* into an IsalChem string *w*. The example corresponds to isobutylamine. It can be seen that the algorithm optimizes the number of pointer movement instructions required to insert the required structures into the temporary graph *G*, which results in a shorter resulting string *w*. Another example of a trace of the execution of the conversion algorithm is provided in the Supporting Information.

**Figure 16:**
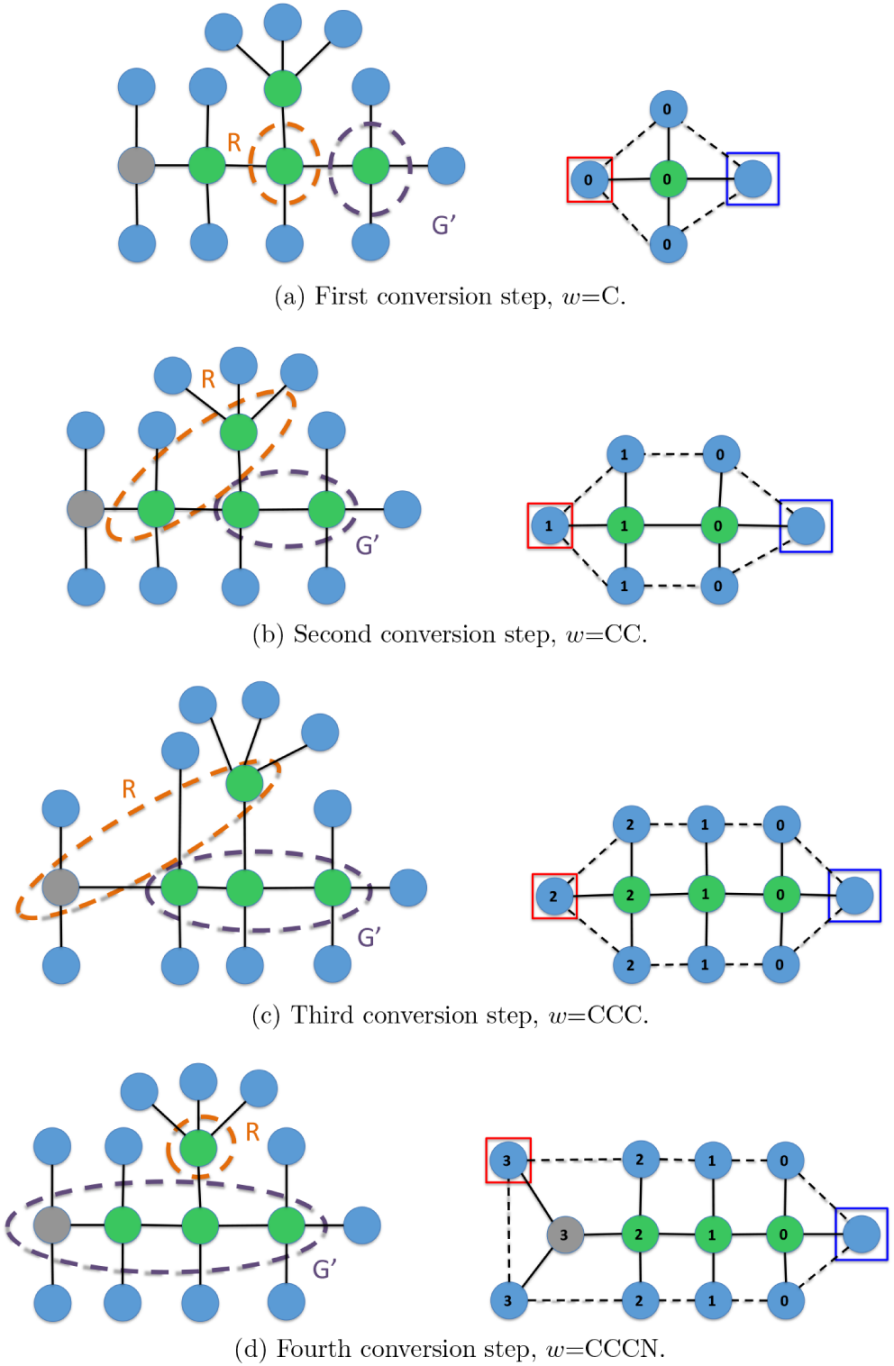
First part of the conversion of the isobutylamine graph into the string CCCN ++C. The input graph *M* is shown on the left, while the temporary graph *G* is shown on the right. The subgraph *G*’ of the non-hydrogen atoms of *G* and the set of waiting non-hydrogen atoms *R* are marked with dashed ellipses on the left.

**Figure 17:**
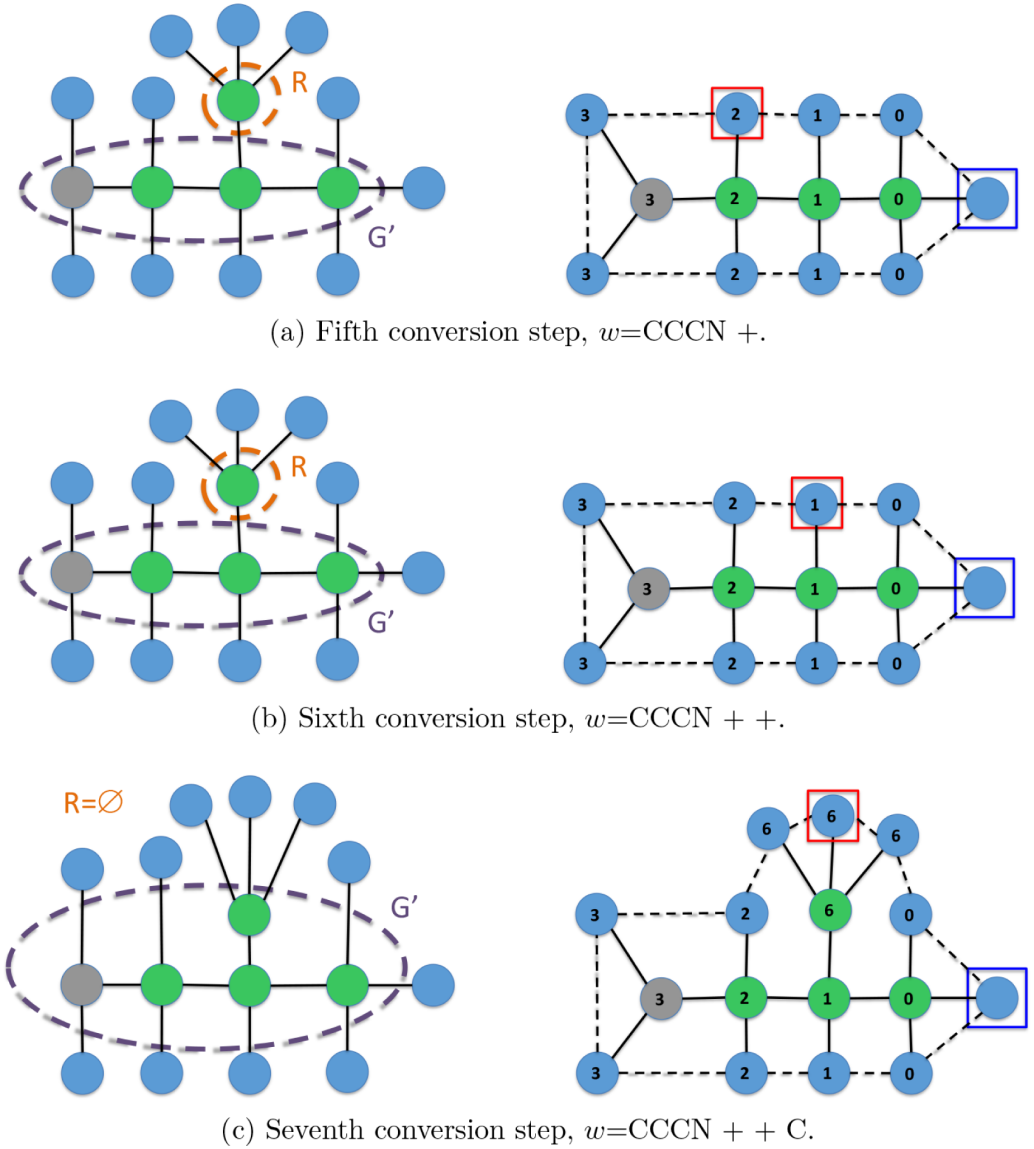
Second part of the conversion of the isobutylamine graph into the string CCCN ++C. The input graph *M* is shown on the left, while the temporary graph *G* is shown on the right. The subgraph *G*’ of the non-hydrogen atoms of *G* and the set of waiting non-hydrogen atoms *R* are marked with dashed ellipses on the left.

## Properties

The desirable properties of the proposal are discussed next. One of the key features of the proposal is that all sequences of IsalChem instructions are valid IsalChem strings, each of which corresponds to a molecule. Because of this, any modification to a string still results in a valid molecule, unlike other notations, where there can be changes to a valid string that result in invalid strings.

The alphabet (set of tokens) formed by the IsalChem instructions will be noted Σ, so that the set of all strings on Σ is denoted as Σ*. There are some distance metrics that can be employed on Σ*.^26^ Among them, the Levenshtein distance is the most widely used one. For example, it is used to measure the dissimilarity between two DNA sequences. The Levenshtein distance *d*(*w*_1_,*w*_2_) between two strings *w*_1_, *w*_2_ ∈ Σ* is the minimum number of edits (insertions, deletions, or substitutions) required to transform *w*_1_ to *w*_2_. Formally, Levenshtein distance *L*_*d*_ between two strings *a* and *b* can be defined as follows, with |*x*| meaning the number of symbols in the string *x*, *H*(*a*) meaning the head of *a* (i.e., the first symbol of *a*) and *T*(*x*) meaning the tail of *x* (i.e., the substring resulting from removing the first symbol from *x*):

$$L_{d}\left(a,b\right)=\left\{ \begin{array}{cc} \left|a\right| & \mathrm{if}\left|b\right|=0\\ \left|b\right| & \mathrm{if}\left|a\right|=0\\ L_{d}\left(T\left(a\right),T\left(b\right)\right) & \mathrm{if}H\left(a\right)=H\left(b\right)\\ 1+\min\left\{L_{d}\left(T\left(a\right),b\right),L_{d}\left(a,T\left(b\right)\right),L_{d}\left(T\left(a\right),T\left(b\right)\right)\right\} & \mathrm{otherwise} \end{array}\right.$$

The four properties that characterize a distance are fulfilled by Levenshtein distance:

- Non-negativity: *d*(*w*_1_,*w*_2_) ≥ 0
- Identity: *d*(*w*,*w*) = 0
- Symmetry: *d*(*w*_1_,*w*_2_) = *d*(*w*_2_,*w*_1_)
- Triangle inequality: *d*(*w*_1_,*w*_3_) ≤ *d*(*w*_1_,*w*_2_) + *d*(*w*_2_,*w*_3_)

Levenshtein distance can be constructively computed, i.e., a minimum length sequence of intermediate strings between *w*_1_ and *w*_2_ can be readily obtained. This allows interpolation of the molecules represented by their respective IsalChem strings. Moreover, each string has a finite set of strings at unit Levenshtein distance. Therefore, for each molecule, there is a finite set of immediate neighbor molecules.

Due to the design of the IsalChem set of instructions, small distances between IsalChem strings translate into small differences between their represented molecules. That is, in most cases, similar strings represent similar molecules. This comes from the simplicity of the instructions and the way that pointer updates are managed. Each time a pointer is updated, it is kept in the same region of the graph. In essence, pointer moves are always relative to the previous pointer position. This implies that a substring of instructions will generate the same molecular substructure no matter where the substring occurs in the overall string.

Moreover, if one instruction is changed to another, then the subsequent insertion instructions will generate the same molecular substructure but attached to a different insertion point. An IsalChem instruction can move at most one pointer and, at most, one step on the doubly linked list *L*. This implies that pointer movements keep the pointers within the same region of the molecule.

Other provisions aim to avoid sharp changes. The hydrogen atoms are inserted and removed at the previous location of the pointers so that the structure of the doubly linked list *L* is preserved as much as possible. Instructions that insert an atom with a nonsingle bond fall back gracefully to their single bond versions in case the current graph does not allow the nonsingle bond insertion. Consequently, an atom of the specified element is still inserted.

Also, the design of the instruction set guarantees that each instruction inserts at most one non-hydrogen atom. Therefore, each unit of Levenshtein distance translates into, at most, one inserted, deleted, or substituted non-hydrogen atom on the represented molecule. This provides an upper bound for the number of non-hydrogen atoms in a molecule, i.e., a string that contains *n* tokens represents a molecule with at most *n* non-hydrogen atoms.

The instruction set is designed to produce the minimal possible change in the IsalChem state for each step, and it is also aimed at keeping the changes local to the current position of the primary pointer. Local sequence alignment on two IsalChem strings is expected to reveal many similar or identical substructures/motifs. The instructions with the highest potential to disturb this alignment are the join instructions J/J2 because the primary and secondary pointers may be far apart in the molecular structure. On the contrary, any substring that does not contain J nor J2 is guaranteed to generate the same subgraph, no matter where it appears within an IsalChem string. Therefore, locality and composability hold for the substrings that do not involve the secondary pointer.

Since all strings in Σ* are valid, it is extremely easy to randomly sample strings, which corresponds to a random sampling of their associated molecules. First, the length of the string is randomly generated. Then, the tokens of the string are randomly chosen among the IsalChem instructions. The probability distributions employed for such generation can be tuned depending on the requirements of the application at hand.

The presented proposal has the potential to be extended to support large macromolecules. This extension is left for future work. Taking the BigSMILES framework as the starting point,^27^ a similar syntax could be defined. The IsalChem set of instructions could be augmented with different instruction types to mark connection points within an IsalChem string. In this way, the specific location of each connection point in the associated IsalChem graph would be defined. A polymer would be specified by several IsalChem strings, one for each possible repeating unit. Fragment name definitions could also be handled, so a significant name is given to an IsalChem string to employ the name as a shorthand for the string.

## Experiments

A set of computational experiments has been carried out in order to examine the characteristics of our proposal. First of all, the topological structure of the IsalChem language is considered. After that, the molecular similarity of the compounds represented by nearby IsalChem strings is checked. Finally, the length of the IsalChem strings is compared to the standard SMILES notation. All experiments were developed using the Python programming language and the RDKit library.

### Topological Structure

In this subsection, two experiments are carried out to ascertain the topological properties of the IsalChem language. Levenshtein distance between IsalChem strings (Properties section) defines a topology on the IsalChem language. That is, each pair of IsalChem strings is at a certain Levenshtein distance, so the immediate neighbors of an IsalChem string are those strings at unit Levenshtein distance. Moreover, Levenshtein distance is constructive, i.e., the shortest path between any pair of IsalChem strings can be computed. The shortest path is a sequence of IsalChem strings that goes from the first string to the last string in steps of unit Levenshtein distance. In other words, the insertion, modification, or deletion of a single token is done from one string to the next string in the shortest path. Consequently, Levenshtein distance defines a topology on the IsalChem language.

The first topology experiment showcases the immediate neighborhood of a molecule. The propane molecule (Figure 18a) has been chosen for this purpose. The number of immediate neighbors of propane is 142, so Figure 18 shows just 8 of them. As can be seen, all the immediate neighbors have common structures with propane. Among the neighbors there are some with less atoms (Figure 18c,e,f and h), other with the same number of atoms (Figure 18d), and finally some of them have more atoms (Figure 18b,g and i). Therefore, the set of immediate neighbors contains molecules that are very similar to propane, but with slightly different complexities.

**Figure 18:**
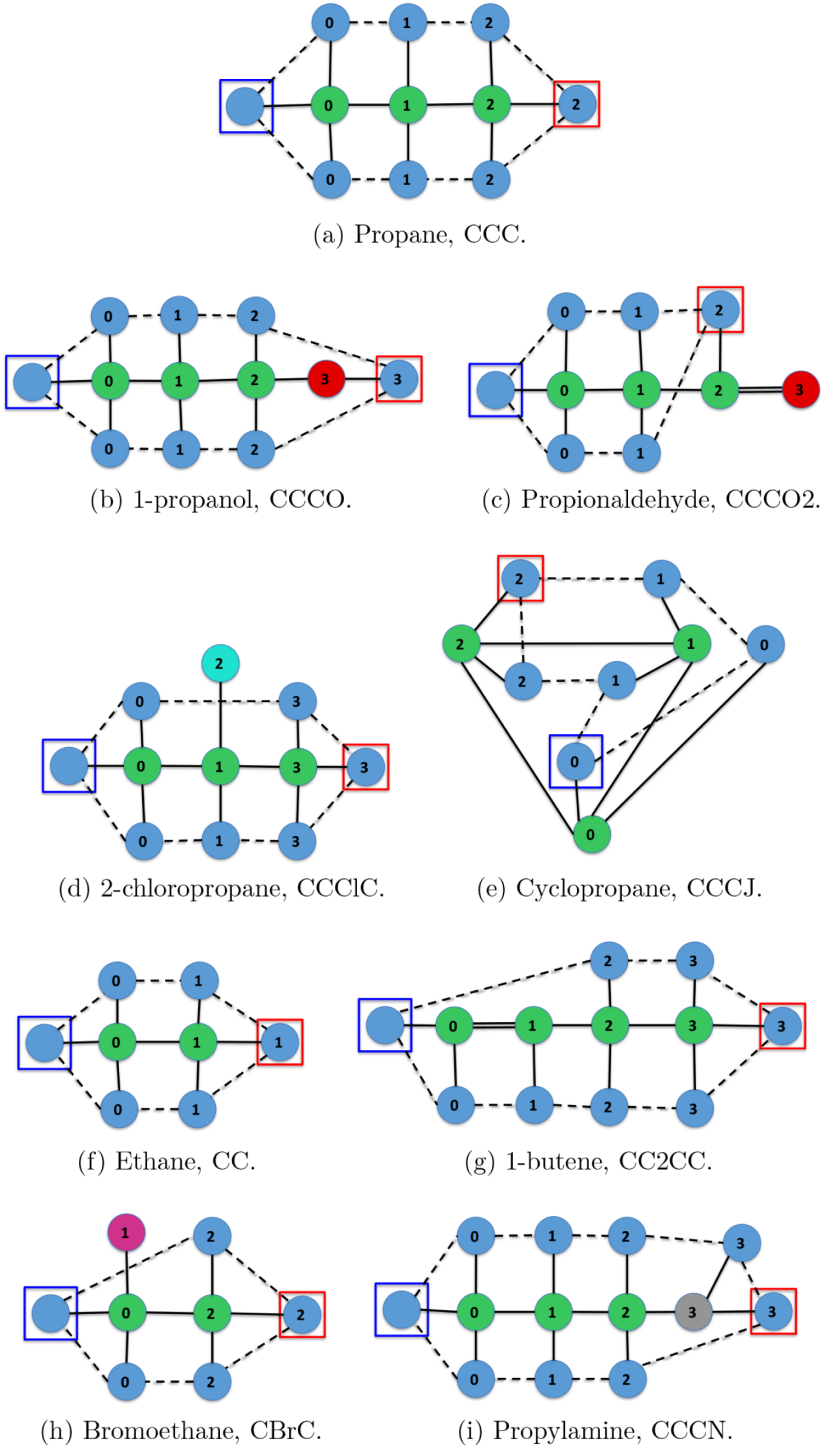
Propane molecule (top row) and some of its immediate neighbor molecules at unit Levenshtein distance (following rows).

The second topology experiment illustrates an example of the shortest path according to the Levenshtein distance between two IsalChem strings. The 2-chloro-2-hydroxyethanal molecule has been chosen as the start molecule, while the 4-bromopentanal molecule has been selected as the final molecule. It turns out that the Levenshtein distance between them is 5, i.e., the minimum number of edits (insertion, modification, or deletion of a single token) to go from 2-chloro-2-hydroxyethanal to 4-bromopentanal is 5. Figure 19 shows the six molecules that lie in the shortest path. The first edit (from Figure 19a,b) is an insertion; O2 is inserted at the end. The second edit (from Figure 19b,c) is a modification; the O is changed to a C. In this particular case, all five edits are insertions or modifications. A simple example of a shortest path with deletions is the reverse path, i.e., the shortest path from 4-bromopentanal to 2-chloro-2-hydroxyethanal. Please note that the insertions in the forward path turn into deletions in the reverse path. As seen, the changes in the molecular structure are small as an edit is performed along the shortest path.

**Figure 19:**
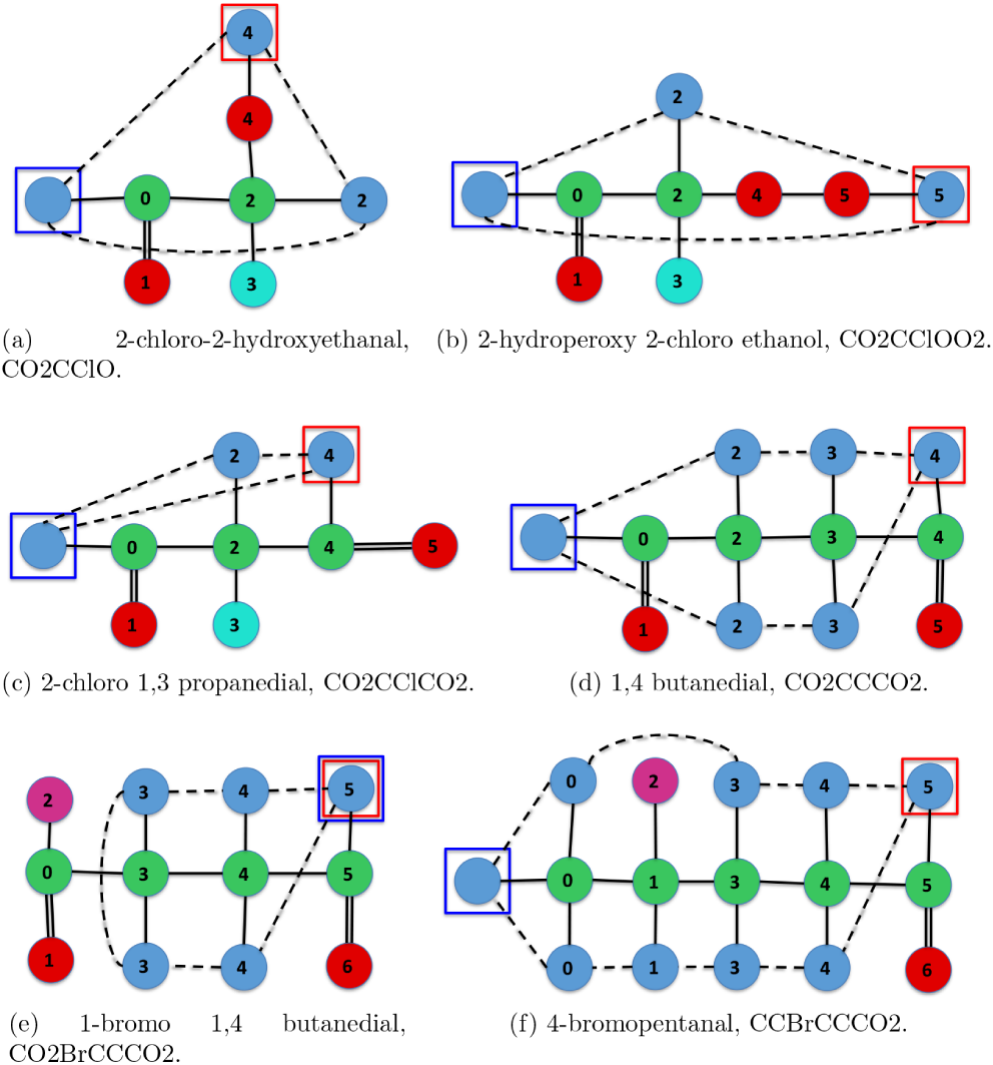
Shortest path according to Levenshtein distance from the 2-chloro-2-hydroxyethanal molecule to the 4-bromopentanal molecule.

Together, these two topological experiments demonstrate that the IsalChem language, equipped with the Levenshtein distance, has a meaningful topological structure. That is, the immediate neighbors of a molecule are pretty similar to it, while the shortest paths can be computed to go from one string to another by taking steps that change the molecular structure progressively.

At this point, it is worth making a comparison with the SELFIES notation,^19^ which does not contain invalid strings. SELFIES processes an input string according to a table of derivation rules that, given the current internal state of the algorithm and the current input symbol, transitions to another internal state and performs an operation, such as the insertion of an atom into the graph of the molecule under construction. However, if the current input symbol is unexpected at the current internal state, then the results do not make sense. More unexpected input symbols may follow, i.e., an offending substring may occur, leading to even more strange behavior. In most cases, these offending substrings are ignored by SELFIES, which inevitably leads to a serious failure of topological structure in the set of SELFIES strings. In other words, nearly identical SELFIES strings often correspond to very different molecules because offending substrings have been ignored. Therefore, no useful notions can be defined for the neighbors of a molecule or the shortest path between two molecules. This is a fundamental limitation of the SELFIES notation, which is designed to avoid invalid strings but not to generate a topological structure.

In order to illustrate this fundamental inability of the SELFIES notation to generate a topological structure among strings, an experiment has been carried out using the previous example molecules that were studied under our proposal. Table 3 shows the shortest path in the SELFIES notation from the 2-chloro-2-hydroxyethanal molecule to the 4-bromopentanal molecule. As seen, many intermediate strings include offending substrings. These substrings are ignored by the SELFIES algorithm, so the resulting molecules have strange features, such as the lack of a bromine and/or an oxygen atom that do appear in the SELFIES strings. This prevents any meaningful processing of the shortest path between these strings. Table 4 lists some SELFIES strings that are immediate neighbors of the string representing propane, namely [C]­[C]­[C]. Again, it can be observed that many kinds of offending substrings occur. Important tokens are ignored or, even worse, produce strange results. Therefore, the distance among SELFIES strings can hardly be employed to estimate the similarity among their represented molecules.

**Table 3:** Shortest Path in the SELFIES Notation, According to Levenshtein Distance, from the 2-Chloro-2-Hydroxyethanal Molecule to the 4-Bromopentanal Molecule^a^

SELFIES string	SMILES conversion	Represented molecule
[O][=C][C][Branch1][C][O][Cl]	O=CC(O)Cl	2-chloro-2-hydroxyethanal
[O][=C][C][Branch1][C][O][Cl]**[Br]**	O=CC(O)Cl	2-chloro-2-hydroxyethanal
[O][=C][C][Branch1][C][O][Cl]**[=O][Br]**	O=CC(O)Cl	2-chloro-2-hydroxyethanal
[O][=C][C][Branch1][C][O][C][=O]**[Br]**	O=CC(O)C=O	2-hydroxypropanedial
[O][=C][C][Branch1][C][C][C][=O]**[Br]**	O=CC(C)C=O	2-methylpropanedial
[O][=C][C][Branch1][Branch1][C][C][C][=O][Br]	O=CC(CCC=O)Br	2-bromopentanedial
[O][C][Branch1][Branch1][C][C][C][=O][Br]	OC(CCC=O)Br	1-bromo butan 1-ol 4-al
[C][C][Branch1][Branch1][C][C][C][=O][Br]	CC(CCC=O)Br	4-bromopentanal

a The results of the conversion to SMILES notation are shown in the second column. Offending substrings are marked in bold.

**Table 4:** Some Immediate Neighbors in the SELFIES Notation, According to Levenshtein Distance, for the [C]­[C]­[C] String, which Represents Propane^a^

SELFIES string	SMILES conversion	Represented molecule
[C][#N]**[C][C]**	C#N	formonitrile
[C][C][#N]**[C]**	CC#N	acetonitrile
**[=O]**[C][C][C]	OCCC	propan-1-ol
[C][=O]**[C][C]**	C**=**O	formaldehyde
**[=N]**[C][C][C]	NCCC	propan-1-amine
**[=C]**[C][C][C]	CCCC	butane
[C][Br]**[C][C]**	CBr	bromomethane
[C][C][Br]**[C]**	CCBr	bromoethane
[C][**=**O]**[C]**	C**=**O	formaldehyde
[C][Br]**[C]**	CBr	bromomethane

a The results of the conversion to SMILES notation are shown in the second column. Offending substrings are marked in bold.

### Molecular Similarity

Next, the chemical similarity of the molecules associated with IsalChem strings that are close according to the Levenshtein distance is investigated. To this end, the well-known, freely available ZINC database of compounds has been chosen[Fn ba-fn2]. A random subset of 1,000 molecules has been drawn from ZINC. Each ZINC molecule has been converted to a compressed IsalChem string by Algorithm 3. Then, a discrete probability distribution has been obtained for each molecule, where each IsalChem instruction has a probability of occurrence proportional to its abundance in the IsalChem string. After that, successive random edits (insertions, modifications, or deletions) have been applied to each IsalChem string, according to its discrete probability distribution, to obtain strings that are further and further away according to the Levenshtein distance. The default fingerprints of each molecule from the RDKit library have been computed (the result of applying RDKit fingerprinting to each molecule is a bit vector). Finally, several molecular similarity metrics are calculated from those fingerprints in order to evaluate the similarity of each initial IsalChem string with the strings resulting from the successive random edits (the similarity between two fingerprints is taken as a proxy of the similarity between the corresponding molecules). This way, the progression of molecular similarity as the Levenshtein distance grows is measured. The following similarity metrics have been chosen for evaluation because they are well-established standards: Tanimoto, Dice, cosine, Kulczynski, McConnaughey, Russel, and Sokal. Each of these metrics measures similarity between bit vectors *V*_*a*_ and *V*_*b*_, using |*V*_*i*_| and ∑*V*_*i*_ to mean the length and the number of bits set to 1 in the bit vector *V*_*i*_, respectively, and *V*_*a*_·*V*_*b*_ to mean the bit-wise product of *V*_*a*_ and *V*_*b*_. Each metric is defined as

- Tanimoto similarity: $\mathrm{Tani}\left(V_{a},V_{b}\right)=\frac{\sum \left(V_{a}\cdot V_{b}\right)}{\sum V_{a}+\sum V_{b}-\sum \left(V_{a}\cdot V_{b}\right)}$

- Dice similarity: $\mathrm{Dice}\left(V_{a},V_{b}\right)=\frac{2\cdot \sum \left(V_{a}\cdot V_{b}\right)}{\sum V_{a}+\sum V_{b}}$

- Cosine similarity: $\mathrm{Coss}\left(V_{a},V_{b}\right)=\frac{V_{a}\cdot V_{b}}{\sqrt{\left|V_{a}\right|+\left|V_{b}\right|}}$

- Kulczynski similarity: $\mathrm{Kulc}\left(V_{a},V_{b}\right)=\frac{\left(\sum V_{a}+\sum V_{b}\right)\cdot \sum \left(V_{a}\cdot V_{b}\right)}{2\cdot \left(\sum V_{a}\right)\cdot \left(\sum V_{b}\right)}$

- McConnaughey similarity: $\mathrm{McCo}\left(V_{a},V_{b}\right)=\frac{\left(\sum V_{a}+\sum V_{b}\right)\cdot \left(\sum V_{a}+\sum V_{b}\right)-\left(\sum V_{a}\right)\cdot \left(\sum V_{b}\right)}{\left(\sum V_{a}\right)\cdot \left(\sum V_{b}\right)}$

- Russel similarity: $\mathrm{Russ}\left(V_{a},V_{b}\right)=\frac{\sum \left(V_{a}\cdot V_{b}\right)}{\left|V_{a}\right|}$

- Sokal similarity: $\mathrm{Sokal}\left(V_{a},V_{b}\right)=\frac{\sum \left(V_{a}\cdot V_{b}\right)}{2\cdot \sum V_{a}+2\cdot \sum V_{b}-3\cdot \sum \left(V_{a}\cdot V_{b}\right)}$

Figure 20 shows the results of this experiment for Tanimoto similarity. The results for the rest of the similarity measures are similar and are available as Supporting Information. As seen, the overall trend is the same for all computed molecular similarity measures. The similarity metrics decrease smoothly as the Levenshtein distance among IsalChem strings increases. These results demonstrate that the topology among the strings in the IsalChem language translates into molecular similarity.

**Figure 20:**
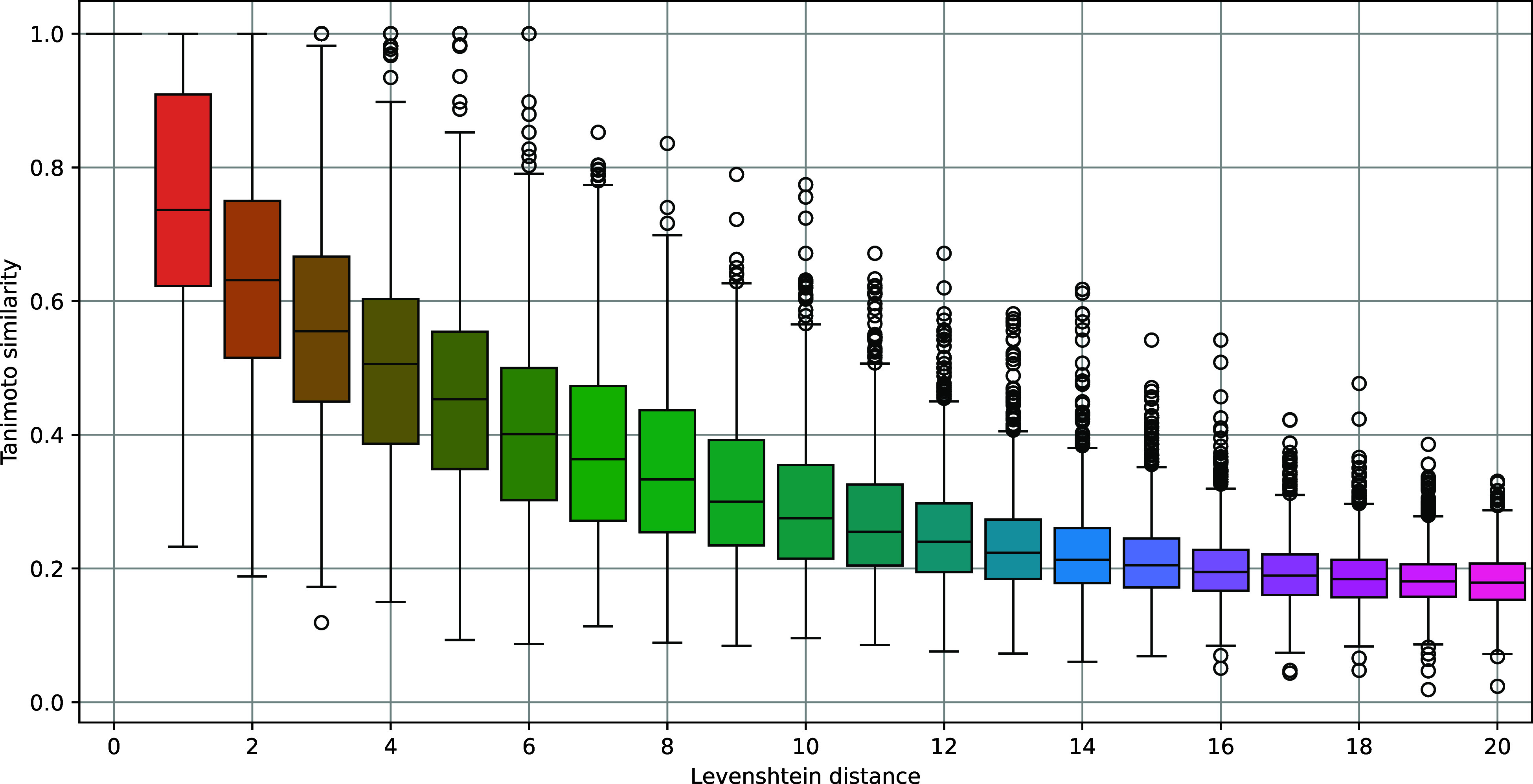
Boxplots of Tanimoto similarity versus Levenshtein distance, for IsalChem strings obtained by successive random edits of IsalChem strings associated with molecules from the ZINC data set. Please note that zero Levenshtein distance leads to identical molecules and, hence, unit Tanimoto similarity.

### Representation Length

This experiment is devoted to the evaluation of the length of IsalChem strings. The reference standard is the length of the corresponding SMILES string. The same setup as in the molecular similarity experiment has been considered. That is, a random subset of 1,000 molecules has been drawn from ZINC. Then, each ZINC molecule has been converted to a compressed IsalChem string by Algorithm 3. After that, the number of tokens of the SMILES and IsalChem strings of each of the 1,000 molecules has been determined. In addition to this, the same procedure has been followed for the SELFIES and InChI notations for comparison purposes. Therefore, the same 1,000 molecules have been represented with SELFIES and InChI, and their number of tokens has been calculated.

Figures 21–23 show the result of the experiment for IsalChem, SELFIES and InChI, respectively. It can be observed that the lengths of the tested notations are strongly correlated, with an approximate linear dependency. The equation of the best-fit regression line for IsalChem is

$$Length_{IsalChem}=1.31\cdot Length_{SMILES}-6.68$$

with a coefficient of determination *R* ^2^ = 0.758. In other words, on average IsalChem strings are longer than SMILES strings by less than 31%. Given the current computer storage capacity, this is a very small overhead.

**Figure 21:**
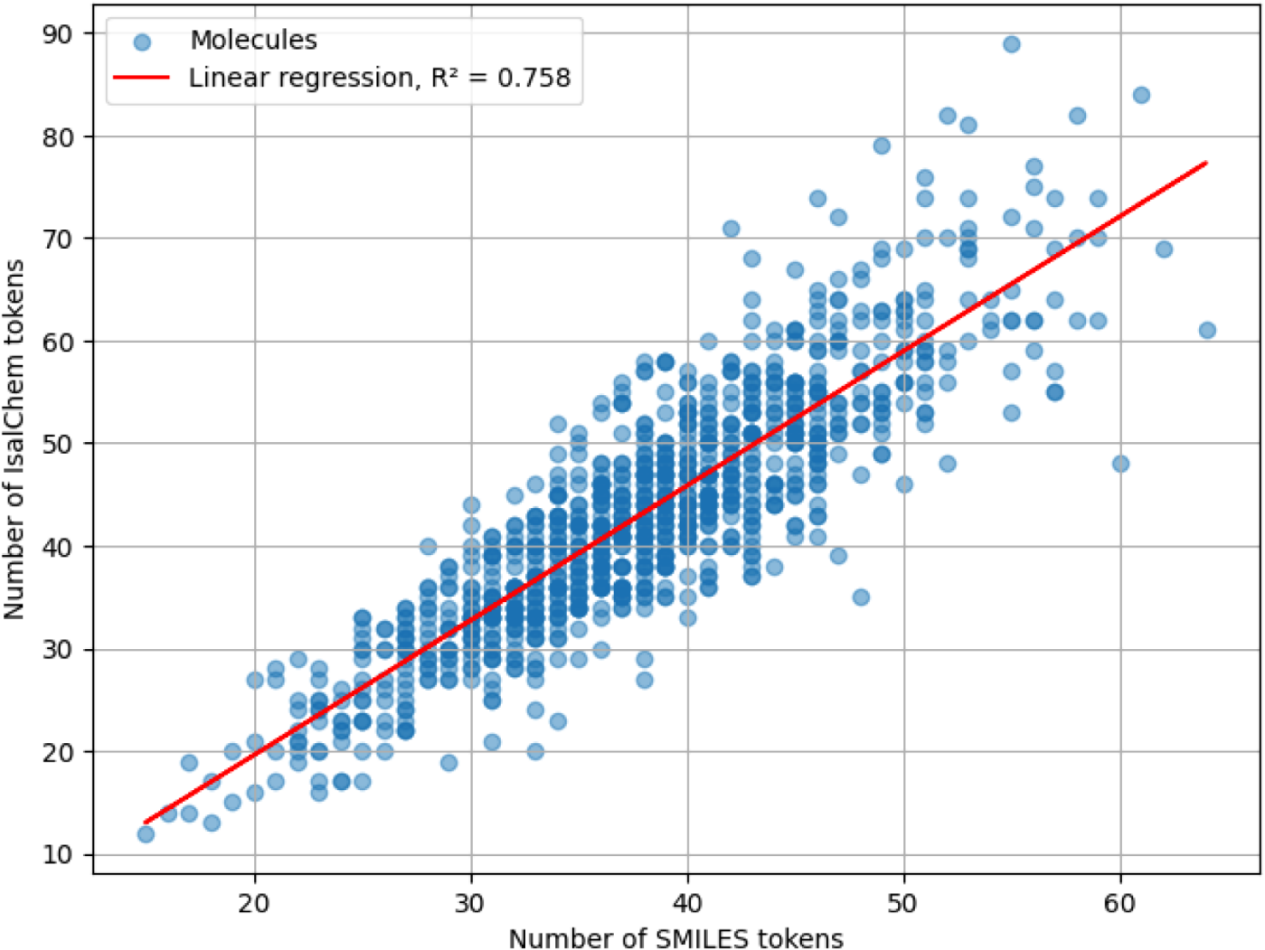
Scatterplot of the length of SMILES and IsalChem strings, measured in numbers of tokens, for molecules from the ZINC data set. The result of the linear regression of the data is also shown.

**22:**
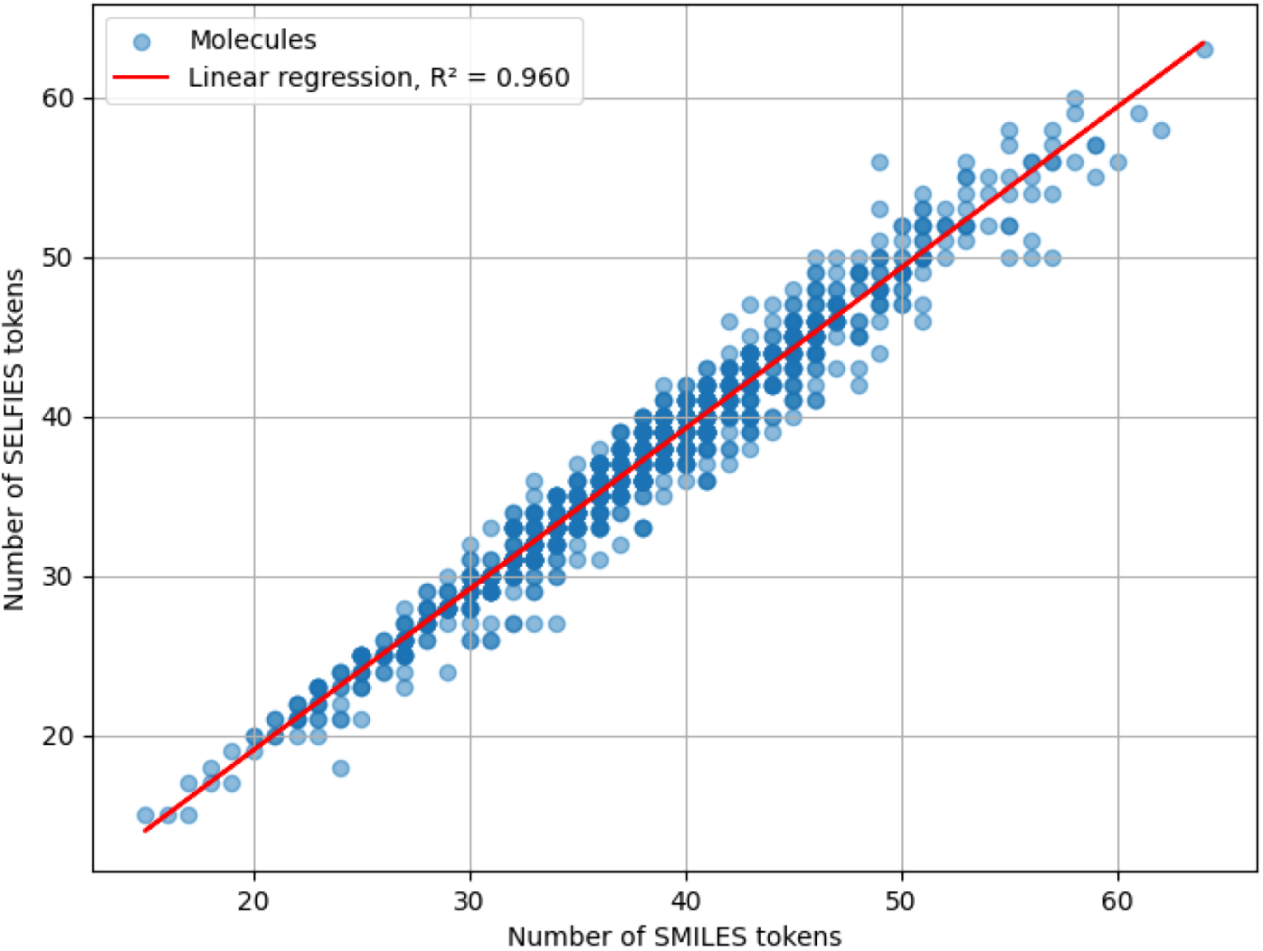
Scatterplot of the length of SMILES and SELFIES strings, measured in numbers of tokens, for molecules from the ZINC data set. The result of the linear regression of the data is also shown.

**Figure 23:**
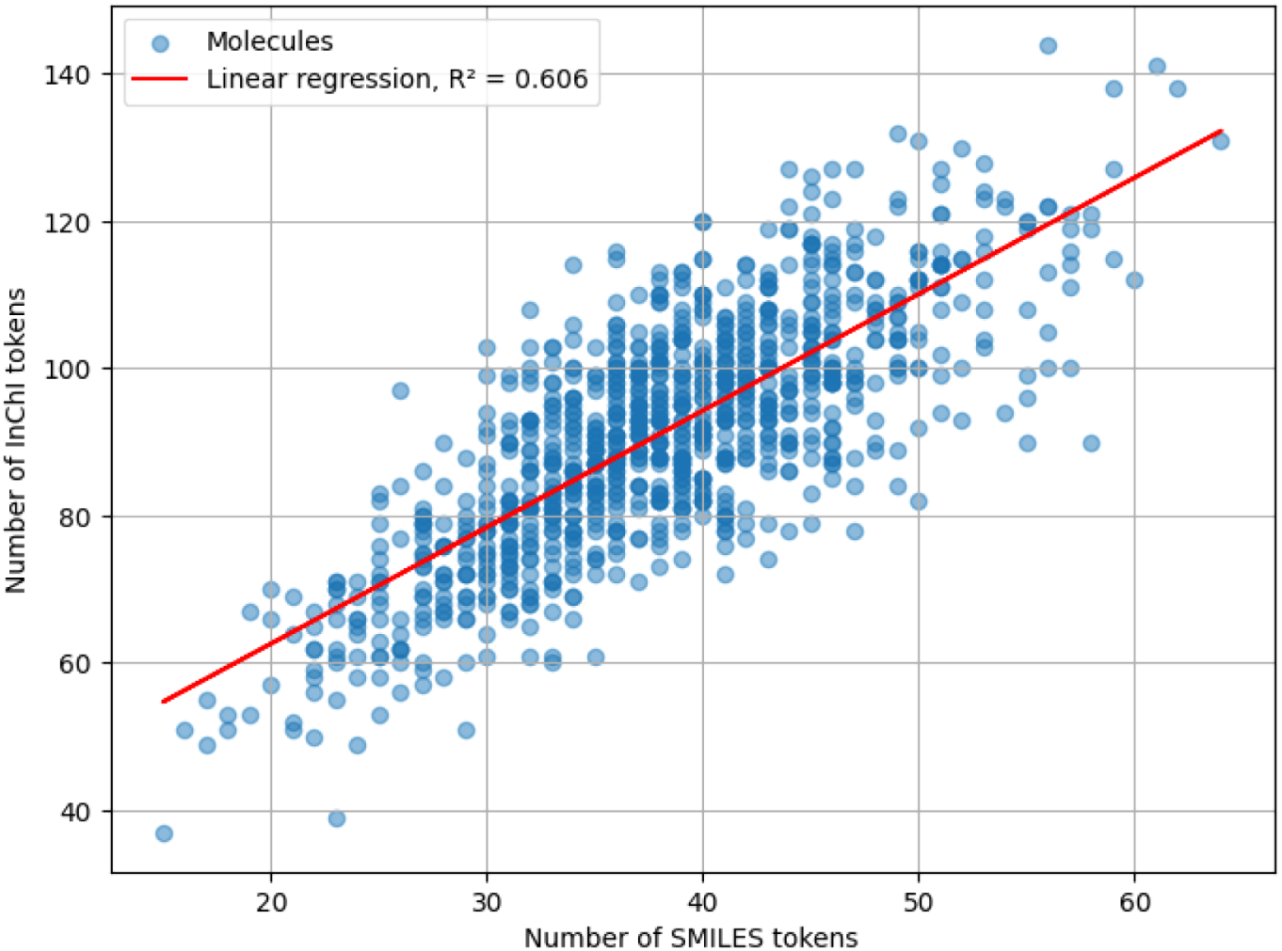
Scatterplot of the length of SMILES and InChI strings, measured in numbers of tokens, for molecules from the ZINC data set. The result of the linear regression of the data is also shown.

The best-fit regression lines for SELFIES and InChI are

$$Length_{SELFIES}=1.01\cdot Length_{SMILES}-1.16$$

$$Length_{InChI}=1.58\cdot Length_{SMILES}+30.97$$

with coefficients of determination 0.96 and 0.606, respectively. As seen, the lengths of SELFIES strings exhibit a nearly perfect correlation with SMILES strings. On the other hand, InChI strings are the longest among the four tested notations, with a weaker correlation to SMILES string length.

Some insight about the structure of the representation systems of SMILES and IsalChem can be gained by examining two examples of molecules with large differences in the length of their representations. One of the molecules is represented in this way:

$$\begin{array}{l} N\#\mathrm{Cc}1\mathrm{nn}\left(C\left(N\right)=O\right)c\left(N\right)c1C\#N\\ \mathrm{NCCN}3\mathrm{CCN}3\mathrm{CN}+\mathrm{NCO}2\mathrm{N‐‐J} \end{array}$$

with string lengths of 24 and 17 tokens, respectively. The other molecule is represented as follows:

$$\begin{array}{l} \mathrm{COC}\left(C\right)\left(C\right)\mathrm{CNC}\left(=O\right)\mathrm{COC}1\mathrm{CCCC}1\\ \mathrm{COCC}+\mathrm{NCCC‐‐OC‐‐‐‐‐O}2‐C\mathrm{CCCJ‐‐‐‐‐‐‐C} \end{array}$$

with string lengths of 25 and 33 tokens, respectively.

From the above examples, it can be noticed that SMILES employs extra tokens for the specification of branches. Conversely, IsalChem spends additional tokens in order to move the pointers to the location of the next atom insertion. Depending on the relative importance of these factors for a particular molecule, one notation is shorter than the other.

### Token Prediction Benchmark

A benchmark comparison of the SMILES, SELFIES, InChI, and IsalChem notations has been conducted. The experimental methodology of ref 28 has been considered. In particular, the LSTM and GRU models have been employed, with embedding dimensions 4, 8, 16, and 32; and hidden sizes 8, 16, 32, and 64. A training set of 10,000 samples randomly drawn from the ZINC data set has been used with an 80%/20% training/validation split. Each model has been trained for 1,000 epochs with a batch size of 64 samples. The task is to predict a token, randomly chosen from a string representing a ZINC molecule in the notation at hand. It is also measured how often the predicted token is not the correct one but forms a valid string. Finally, some molecular similarity metrics are computed for the valid strings, as compared with the correct strings.

Tables 5 and 6 report the benchmark results for LSTM and GRU models, respectively. Both kinds of models exhibit similar behavior. IsalChem attains the best results in validation model loss, correct prediction, and validity. InChI is the worst model in these three metrics, while it is the best according to the molecular similarity measures. This suggests that the InChI notation is so strict in its syntactic rules that, in many cases, the predicted token leads to an invalid string; conversely, for the few valid strings, the similarity with respect to the original one is usually high. In this respect, the only notation that is a fair comparison with IsalChem is SELFIES, since both guarantee 100% validity. It can be observed that IsalChem exhibits better molecular similarity results than SELFIES according to all considered molecular similarity measures. Therefore, it can be concluded that IsalChem shows a very high performance for this model learning task, as compared with the other three notations.

**Table 5:** Results of the Token Prediction Task for LSTM Recurrent Models^a^

Metric	SMILES	SELFIES	InChI	IsalChem
Model size	(8, 16)	(4, 8)	(32, 64)	(4, 8)
Model loss	1.380	1.582	5.404	1.049
Correct	60.462	56.356	47.478	68.990
Valid	71.594	100.000	50.912	100.000
Tanimoto	0.963	0.786	0.992	0.849
Dice	0.978	0.844	0.995	0.895
Cosine	0.978	0.845	0.995	0.895
Kulczynski	0.978	0.846	0.995	0.895
McConnaughey	0.955	0.691	0.989	0.790
Russel	0.021	0.018	0.021	0.019
Sokal	0.943	0.729	0.990	0.804

a The best model size is reported in the first row, in the format (embedding dimension, hidden size). The second row indicates the final value of the validation cross-entropy loss of the best network (lower is better). The correct prediction and validity ratios are reported in the third and fourth rows (higher is better). Some molecular similarity measures are reported in the last rows (higher is better).

**Table 6:** Results of the Token Prediction Task for GRU Recurrent Models^a^

Metric	SMILES	SELFIES	InChI	IsalChem
Model size	(8, 16)	(4, 8)	(32, 64)	(4, 8)
Model loss	1.413	1.449	4.585	0.967
Correct	59.834	55.816	47.451	68.860
Valid	71.001	100.000	50.739	100.000
Tanimoto	0.963	0.784	0.992	0.847
Dice	0.978	0.843	0.994	0.893
Cosine	0.978	0.843	0.994	0.893
Kulczynski	0.978	0.844	0.994	0.894
McConnaughey	0.956	0.688	0.989	0.787
Russel	0.021	0.018	0.022	0.019
Sokal	0.944	0.725	0.990	0.802

a The best model size is reported in the first row, in the format (embedding dimension, hidden size). The second row indicates the final value of the validation cross-entropy loss of the best network (lower is better). The correct prediction and validity ratios are reported in the third and fourth rows (higher is better). Some molecular similarity measures are reported in the last rows (higher is better).

### De Novo Drug Design

A short analysis has been carried out concerning the potential of IsalChem to generate molecules for the field of de novo drug design. Following one of the experiments carried out in ref 29, a simple molecule generation model without training is employed. The SMILES, SELFIES, InChI, and IsalChem notations have been considered. The 10,000 molecules subset of the ChEMBL data set chosen in ref 29 has been used. For each of the considered notations and each molecule, a token has been chosen at random from the representation of the molecule, and it has been replaced by another token according to a token probability distribution that follows the relative abundances of the tokens in the ChEMBL subset. Then, the resulting fractions of valid, unique, and novel molecules have been computed. Also, the original and generated molecular distributions have been compared according to the Fréchet ChemNet distance and the Kullback–Leibler divergence (KLD) computed over several molecular descriptors.

Table 7 lists the obtained results. It can be noticed that IsalChem obtains the best results in validity, uniqueness, and novelty. These results suggest that a small change in a string leads to a small but relevant change in the represented molecule. Of course, all strings are valid. The Fréchet ChemNet distance (FCD) is highest for IsalChem, which is an additional indication of the rich variety of molecular variations that can be generated. Conversely, the SELFIES notation also has a validity score of 100% but a much smaller variety as seen from its lower FCD. SMILES and InChI attain a very low validity score since their design includes strong syntactic constraints that invalidate many possible strings. The KLD values do not show any specific trend, except for the much higher values for InChI. This suggests that among the tiny fraction of valid InChI strings, large variations exist with respect to the original molecular distribution.

**Table 7:** Results of the Molecule Generation Experiment for the ChEMBL Dataset^a^

Metric	SMILES	SELFIES	InChI	IsalChem
Valid	0.1360	1.0000	0.0045	1.0000
Unique	1.0000	0.9940	1.0000	1.0000
Novel	0.7860	0.9562	0.5909	1.0000
FCD	3.4731	4.1640	37.3184	42.4452
KLD—MolWt	0.0294	0.0476	0.3124	0.0073
KLD—HeavyAtomMolWt	0.0310	0.0486	0.3123	0.0021
KLD—MolLogP	0.0236	0.0275	0.6241	0.0843
KLD—NumRotatableBonds	0.0237	0.0180	0.5541	0.0290
KLD—TPSA	0.0281	0.0125	0.5564	0.0040

a The first three rows list the fractions of valid, unique, and novel molecules. FCD stands for the Fréchet ChemNet Distance. KLD stands for the Kullback–Leibler divergence.

## Discussion

In this section, the main results of our investigation are discussed. The starting point of our approach is the definition of an instruction set and the associated IsalChem language for the representation of molecules. IsalChem has three key characteristics. First of all, it is a regular language that contains all possible strings that can be formed with instructions from the instruction set. In other words, there are no invalid strings, i.e., all strings correspond to a valid molecule. Consequently, all inconveniences and errors that come from the management of invalid strings are entirely removed. Second, this property is achieved without a large increase in the length of the strings, as compared with the standard SMILES notation. This implies that there is no heavy overhead in using the proposed notation instead of SMILES.

Last but not least, Levenshtein distance among IsalChem strings strongly correlates with chemical similarity. That is, strings that have a small Levenshtein distance exhibit strong chemical similarities, as measured by several standard molecular similarity metrics. Moreover, Levenshtein distance defines the shortest path within the set of IsalChem strings, where each step in the path is associated with a small change in the chemical characteristics of the intermediate molecules. Also, it is worth noting that Levenshtein distance and its associated shortest path can be efficiently computed. Consequently, the topology in the IsalChem language is meaningful from a chemical point of view.

There is interest in the use of machine learning (for example, evolutionary algorithms) and deep learning models (for example, large language models) to generate novel molecular designs. These models typically operate by proposing modifications to other molecular designs expressed in some notation, but their performance can be greatly hindered because most changes to valid molecular representations result in invalid strings that do not represent any molecular design.

The topological structure properties of IsalChem are a significant novelty with respect to previous notations. In particular, the SELFIES notation is designed to avoid invalid strings but not to generate a topological structure in the set of strings, as demonstrated in the experimental section above. In other words, the SELFIES notation is not suitable for similarity preservation by the string representation of the molecules. Therefore, its practical applicability is reduced, as compared to the IsalChem notation.

The instructions that move the pointer (+, −, <, and >) iterate through the hydrogen atoms linked to the ordered bonds of each carbon atom. Consequently, isomerism can be managed by including the appropriate pointer movement instructions. This way, the exact carbon bond where a new atom should be inserted is precisely specified. Our approach has the advantage of not needing any specialized tokens to manage stereochemistry. Any three-dimensional arrangement can be specified by pointer movement instructions. This is in contrast to other notations: SMILES requires special tokens, while InChI employs a special sublayer with sublayers and descriptors.

Regarding the computational aspects of processing molecular representation, our approach is equipped with a complete set of algorithms. The basic algorithm is the one that translates an IsalChem string to a molecular graph. An algorithm to convert a molecular graph into an IsalChem string has also been proposed. Furthermore, a compression algorithm has been detailed so that the length of IsalChem strings is kept as short as possible. All these algorithms have been extensively tested in the presented experiments.

Together, these properties establish the IsalChem framework as a chemically significant and computationally efficient alternative to current notations for molecules. Optimization of molecules and exploration of the set of possible molecules may be substantially enhanced by using IsalChem as the reference representation. Any task that requires a fixed string length can be easily accommodated by our proposal by using no-operation instructions.

The design choice for aromatic rings is explained next. One of the design principles of IsalChem is that no invalid strings must exist. Lowercase letter nomenclature for aromatic atoms (such as in SMILES) would clash with this principle. For example, the string “c” would be invalid because it is impossible to have a molecule that only contains “one aromatic carbon”. If a specific notation for aromatic rings were required, it would be more advantageous to have a specific instruction that inserts the complete aromatic ring in one single step. This would keep all strings valid. However, we have not done so because it would defeat the principle that each IsalChem instruction must carry out the smallest possible modification on the molecular structure.

## Conclusions and Future Works

A new method to represent chemical compounds by strings of characters has been proposed. It is based on an instruction set to build the molecular graph step by step. Under this representation, all strings are valid, and small changes in a string correspond to small modifications in the chemical properties of the represented molecule. The shortest path between two strings can be readily computed, which corresponds to a sequence of small changes in chemical structure. Conversion algorithms have been proposed, from string to molecular graph and vice versa. Furthermore, a string compression algorithm has also been developed. This opens the path for more efficient applications of computational intelligence methods to processing chemical information.

Directions for future work are detailed next. The definition of the language will be completed in order to accommodate all details of molecular graphs. There will be a different insertion instruction for each element and for each possible valence of the element. Electric charges associated with specific atoms will also be managed. Furthermore, the proposed symbolic language will be used in conjunction with generative deep models and evolutionary algorithms to infer new molecular structures.

## Data Availability

The demonstration software of IsalChem and associated scripts used to generate molecular structures and their corresponding properties in a machine-readable format are publicly available at https://zenodo.org/records/14997207?token=eyJhbGciOiJIUzUxMiJ9.eyJpZCI6ImQ3ODE0YTMxLWRkOTYtNGY5NC04ODdhLTA0NzA4ODIwNzc3OCIsImRhdGEiOnt9LCJyYW5kb20iOiIzMDZmZjExMDE2NjYyZDk4OTI4NTdjMTlkMGMwOGRkMiJ9.kXj7wvKpjyGSWMxRgvp7bOyzCm6iNVxO3g4DavrISa1cpgEuJoKSwftvAOvMjin0CtZYZoms5iZa7XqewyB_Ag. Sample molecular data sets, generated using IsalChem can be accessed from https://tdcommons.ai/generation_tasks/molgen/#zinc. All molecular structures, along with their properties presented in this study, can be generated directly using the provided software and configuration files.

## Footnotes

a The Python source code of the implementation of the proposed algorithms will be published in case of acceptance.

b
https://tdcommons.ai/generation_tasks/molgen/#zinc.
